# Blood-based biomarkers and major adverse cardiac events after percutaneous coronary intervention: a systematic review with narrative synthesis

**DOI:** 10.3389/fcvm.2026.1817332

**Published:** 2026-10-13

**Authors:** Shyamalendu Tripathy, Minesh Patel

**Affiliations:** 1 Department of Pharmacology, School of Pharmacy, Parul University, Vadodara, Gujarat, India; 2 Department of Pharmacology (Pharmacy), Parul University, Vadodara, Gujarat, India

**Keywords:** biomarkers, coronary artery disease, dual antiplatelet therapy, major adverse cardiac events, NT-proBNP, percutaneous coronary intervention

## Abstract

**Background:**

Major adverse cardiac events (MACE) remain common after percutaneous coronary intervention (PCI) despite advances in stent technology and secondary-prevention pharmacotherapy. Blood-based biomarkers have been proposed as tools for individualised risk stratification, but reported prognostic associations vary widely across studies.

**Objectives:**

This review addressed two prespecified and analytically separate questions. The primary (prognostic) question was: among adults undergoing PCI, are elevated concentrations of blood-based biomarkers associated with subsequent MACE and mortality? The secondary (treatment-effect) question was: among adults undergoing PCI, do specified pharmacotherapeutic strategies reduce MACE? The two questions were addressed using separate eligibility criteria, separate effect measures, separate risk-of-bias instruments and separate syntheses; prognostic associations and treatment effects were not pooled together.

**Methods:**

MEDLINE (via PubMed), Embase (via Ovid), Web of Science Core Collection and the Cochrane Central Register of Controlled Trials (CENTRAL) were searched from 1 January 2010 to 28 February 2025, supplemented by ClinicalTrials.gov, the WHO ICTRP, OpenGrey and backward and forward citation searching. Records were restricted to English-language reports. Eligible studies for the primary question enrolled adults (≥18 years) undergoing PCI, measured a blood-based biomarker at a defined time point relative to the index procedure, and reported MACE or mortality with an extractable adjusted or unadjusted effect estimate. Risk of bias was assessed with QUIPS for prognostic factor studies, supported by ROBINS-I, Cochrane RoB 2.0 and AMSTAR 2 as appropriate to design. Random-effects meta-analysis was to use the restricted maximum likelihood estimator for between-study variance with Hartung–Knapp–Sidik–Jonkman confidence intervals, with DerSimonian–Laird estimates as a sensitivity analysis. No analyte met the minimum study count required to trigger pooling (Section 3.5), so no meta-analysis was performed. Pooling was restricted to biomarker–outcome pairs with at least three methodologically comparable primary studies. Prediction intervals accompany every pooled estimate derived from five or more studies. Certainty of evidence was rated using a GRADE framework adapted for prognostic factor reviews.

**Results:**

Forty-two studies were included, comprising 41 primary reports and one systematic review retained for cross-referencing only, together contributing 37,524 participants to the prognostic synthesis. The evidence base was dispersed across 42 distinct analytes: 38 were evaluated in a single study and four in two studies, so no analyte reached the minimum of three studies prespecified for quantitative pooling. Meta-analysis was therefore not undertaken, and the evidence is reported as a structured narrative synthesis. Elevated concentrations of N-terminal pro-B-type natriuretic peptide, high-sensitivity cardiac troponin T, C-reactive protein and its high-sensitivity assay, interleukin-6, the systemic immune-inflammation index and the neutrophil-to-lymphocyte ratio were each reported as associated with increased risk of MACE, consistently in direction but on the basis of one or two studies per analyte. Evidence for microRNA-497-5p and other emerging analytes rested on single small studies and is reported as preliminary. Thirteen studies were rated at high overall risk of bias, seventeen at moderate and twelve at low. Certainty was very low for the 38 single-study analytes and low for the remainder. Treatment-effect evidence identified under the secondary question is reported separately and was not combined with the prognostic synthesis.

**Conclusions:**

Blood-based biomarkers show consistent directional associations with MACE after PCI, but the evidence is too dispersed for quantitative synthesis: no analyte was evaluated in more than two of the included studies. Heterogeneity in assay platform, sampling time, cut-off derivation and endpoint definition compounds this dispersion. These findings support further evaluation of a small number of candidate markers in adequately powered cohorts with standardised measurement, not the incorporation of any biomarker into routine post-PCI decision-making. No included study randomised patients on the basis of a biomarker result, so no biomarker-guided treatment recommendation can be made.

**Systematic Review Registration:**

https://www.crd.york.ac.uk/PROSPERO/view/CRD420251076074, PROSPERO CRD420251076074.

## Highlights

- Elevated NT-proBNP, hs-cTnT, hs-CRP, IL-6, SII and NLR were associated with MACE after PCI, consistently in direction but on the basis of one or two studies each; the analyses did not evaluate whether these markers improve discrimination, calibration or reclassification beyond established risk models.
- Prognostic biomarker associations and pharmacotherapeutic treatment effects were addressed as two separate review questions, with separate eligibility criteria, effect measures, risk-of-bias instruments and syntheses.
- Evidence for the emerging analytes, including the microRNAs miR-497-5p and miR-142-5p, rests on single small studies and is reported as preliminary, not as equivalent in certainty to that for the more frequently studied inflammatory and cardiac stress markers.
- Substantial heterogeneity in assay methods, sampling timing, cut-off values and MACE definitions, and the absence of biomarker-guided randomised trials, preclude immediate clinical implementation.

## 1 Introduction

Coronary artery disease (CAD) remains the single largest contributor to cardiovascular mortality worldwide, and its burden is rising as populations age (1). Older adults develop more extensive and heavily calcified atherosclerotic plaque, and accumulate comorbidities that both raise the risk of CAD and worsen outcomes after revascularisation (2).

Percutaneous coronary intervention (PCI) is a minimally invasive revascularisation technique that restores blood flow to ischaemic myocardium. Since its introduction in the late 1970s it has become a mainstay in the management of acute coronary syndromes (ACS) and of selected patients with chronic coronary syndrome (CCS) (3, 4). A coronary stent — bare-metal (BMS) or, far more commonly, drug-eluting (DES) — is deployed after balloon dilatation to limit vascular recoil and restenosis. Despite advances in stent design, procedural technique and peri-procedural care, a substantial residual risk of major adverse cardiac events (MACE) persists. MACE is the principal composite measure used to judge procedural success and the effectiveness of adjunctive pharmacotherapy, and it is strongly associated with long-term morbidity and mortality (5).

Predicting which patients will experience MACE after PCI remains difficult, particularly in older adults, and the predictors identified in the existing literature differ substantially between studies because of variation in case mix, sampling strategy and endpoint definition (6). This variation is itself a central methodological problem for any synthesis of the prognostic literature, and it motivated the analytical framework adopted here.

### 1.1 Biomarkers in cardiovascular risk stratification

Biomarkers are measurable biological indicators of underlying physiological and pathological states. In principle they can identify patients at elevated risk of post-PCI MACE even when clinical and angiographic findings appear reassuring, and thereby support more individualised management. It is important at the outset to distinguish three separate claims that are frequently conflated in this literature: that a biomarker is associated with outcome; that it improves prediction beyond established risk factors (discrimination, calibration and reclassification); and that measuring it changes management and improves outcomes. These are progressively stronger claims requiring progressively stronger study designs, and the present review is positioned primarily at the first level.

### 1.2 Inflammatory markers

High-sensitivity C-reactive protein (hs-CRP) is among the most extensively validated inflammatory biomarkers. Elevated hs-CRP, whether measured at baseline or persisting after PCI, has been associated with higher MACE incidence and cardiovascular mortality even among patients achieving low-density lipoprotein cholesterol (LDL-C) targets (7, 8).

Interleukin-6 (IL-6), an upstream cytokine, influences vascular inflammation and plaque instability, and elevated concentrations have been associated with worse cardiovascular outcomes after PCI, particularly in ACS cohorts (9, 10). Composite indices such as the systemic immune-inflammation index (SII) and the neutrophil-to-lymphocyte ratio (NLR) act as integrated markers of immune activation and have been associated with MACE and mortality, most consistently in patients with diabetes or chronic kidney disease (11, 12). Inflammatory activation also interacts with coronary physiology and with autonomic tone: in patients with newly diagnosed unstable angina, hs-CRP and low-frequency heart rate variability power were each independently associated with vessel-specific functional ischaemia assessed by quantitative flow ratio, indicating that inflammatory markers operate within a broader neuro-immune context rather than in isolation (13).

### 1.3 Cardiac stress and injury markers

Biomarkers of myocardial stress and injury are foundational to post-PCI risk assessment. N-terminal pro-B-type natriuretic peptide (NT-proBNP) is released in response to ventricular wall stress and volume overload, and persistent post-procedural elevation has been associated with heart failure, readmission and long-term mortality across diverse populations, including older adults and those with left ventricular dysfunction (14–16).

High-sensitivity cardiac troponins (hs-cTnT and hs-cTnI) index myocardial necrosis. Modest post-procedural troponin elevation, even without overt procedural complication, has been associated with subsequent MACE and all-cause mortality (17), and a dose–response meta-analysis reported that troponin I elevation ≥3× the upper reference limit and creatine kinase-MB (CK-MB) elevation ≥1× the upper reference limit were associated with increased mortality (18).

### 1.4 Emerging and novel biomarkers

Several newer analytes have attracted interest for their potential to identify risk at an earlier or subclinical stage. Circulating miR-497-5p, a microRNA implicated in cardiomyocyte apoptosis and remodelling, has been associated with MACE in a single small ACS cohort undergoing PCI (19). Periostin (*Δ*POSTN), an extracellular matrix glycoprotein involved in myocardial fibrosis, showed short-term prognostic value for postoperative cardiac complications in an off-pump coronary bypass population (20). Asymmetric dimethylarginine (ADMA), a marker of endothelial dysfunction and reduced nitric oxide bioavailability, has been linked to restenosis, cardiovascular mortality and MACE (21), and interleukin-38 (IL-38) and kidney injury molecule-1 (KIM-1) have been reported to add prognostic information in early-phase studies (22, 23). In every case the supporting evidence is limited in volume, derives from small single-centre cohorts, and has not been replicated; these markers are therefore treated throughout this review as preliminary and are not accorded the same certainty as repeatedly evaluated markers.

Multi-marker panels and combinations of biomarkers with established clinical scores (for example PRECISE-DAPT or GRACE) may offer more than any single analyte. Notably, the appropriate test of such combinations is not whether each component is individually associated with outcome but whether the combined model improves discrimination, calibration and reclassification relative to the established model. Studies applying that standard — for example a multimodal model incorporating the SII and NT-proBNP alongside diabetes and hypertension, which achieved a C-index of 0.768 in development but 0.633 on external validation (24), and the enrichment of the post-discharge GRACE score with deceleration capacity, which produced a statistically significant but numerically modest integrated discrimination improvement of 1.04% (25) illustrate both the correct analytical approach and the typically modest magnitude of incremental gain.

### 1.5 Pharmacotherapy after PCI

Recurrent coronary events remain common despite improvements in procedural and medical therapy, making secondary prevention central to post-PCI care (26). Dual antiplatelet therapy (DAPT), combining aspirin with a P2Y12 inhibitor, is the cornerstone after PCI for ACS, with agent selection and duration determined by the balance of ischaemic and bleeding risk. Beta-blockers are indicated for impaired left ventricular function or arrhythmia, with more limited benefit where ejection fraction is preserved. High-intensity statins are first-line for lipid management, with ezetimibe or PCSK9 inhibitors added when LDL-C targets are not met, and bempedoic acid available for statin-intolerant patients (27). ACE inhibitors or angiotensin receptor blockers are important in patients with diabetes or heart failure, with angiotensin receptor-neprilysin inhibitors and mineralocorticoid receptor antagonists offering further benefit in selected groups. Glucagon-like peptide-1 receptor agonists (GLP-1 RAs) and sodium-glucose cotransporter-2 inhibitors (SGLT2i) have demonstrated cardioprotective effects with additional metabolic and renal benefits (28, 29).

These pharmacotherapeutic questions are conceptually and analytically distinct from the prognostic question of whether a biomarker predicts outcome. They are addressed in this review as a clearly delineated secondary objective, and the reasons for keeping the two syntheses separate are set out in Section 2.2.

### 1.6 Review questions and objectives

Following peer review, the analytical framework of this review has been restructured to separate two questions that had previously been reported together.

#### 1.6.1 Objective 1 (primary: prognostic factor question)

Among adults undergoing PCI, is an elevated concentration of a blood-based biomarker, measured at a defined time point relative to the index procedure, associated with the subsequent occurrence of MACE or death? The unit of analysis is the biomarker–outcome association; the effect measures are adjusted or unadjusted hazard ratios (HR), odds ratios (OR) or risk ratios (RR) comparing higher with lower biomarker categories, or per-unit/per-standard-deviation increments.

#### 1.6.2 Objective 2 (secondary: intervention question)

Among adults undergoing PCI, do specified pharmacotherapeutic strategies (P2Y12 inhibitor selection and DAPT duration, CYP2C19 genotype-guided antiplatelet therapy, pre-procedural statin loading, GLP-1 RA and SGLT2i therapy) reduce the incidence of MACE relative to comparator strategies? The unit of analysis is the randomised or adjusted comparison between treatment strategies.

These two objectives address different causal questions, are susceptible to different biases, and require different appraisal instruments. Accordingly, they were pursued with separate eligibility criteria (Sections 2.4 and 2.5), separate risk-of-bias assessment (Section 2.10), and separate synthesis (Sections 3.5–3.11 and 3.12 respectively). At no point were prognostic associations and treatment effects combined in a single pooled estimate or a single meta-regression.12.

## 2 Methods

### 2.1 Protocol, registration and reporting guidance

This systematic review and meta-analysis was conducted and reported in accordance with the PRISMA 2020 statement (6). Because the primary objective concerns prognostic factors rather than interventions, reporting was additionally informed by the CHARMS-PF framework for data extraction in prognostic factor reviews and by the REMARK reporting recommendations for prognostic marker studies; the corresponding items are addressed in Sections 2.7 and 2.8. The completed PRISMA 2020 checklist is provided as Supplementary Table S6.

The review protocol was prospectively registered with PROSPERO (CRD420251076074). Deviations from the registered protocol, all of which were introduced in response to peer review, are listed explicitly in Supplementary Table S7 and are identified as *post hoc* wherever the corresponding results are reported. The principal deviations were: (i) formal separation of the prognostic and treatment-effect objectives; (ii) adoption of the restricted maximum likelihood (REML) estimator with Hartung–Knapp–Sidik–Jonkman confidence intervals in place of DerSimonian–Laird as the primary synthesis method; (iii) introduction of a minimum of three studies for any pooled estimate and ten studies for any test of funnel plot asymmetry; and (iv) addition of the sensitivity analyses described in Section 2.15.

### 2.2 Analytical framework and separation of the two objectives

The distinction between a prognostic association and a treatment effect is fundamental to the interpretation of this review. A prognostic factor analysis asks whether a measured characteristic conveys information about future risk in a defined population; it is inherently observational, is estimated from cohorts, and is threatened principally by confounding, selective reporting of cut-offs, and measurement heterogeneity. An intervention analysis asks whether assigning a treatment changes outcome; it is estimated preferentially from randomised trials and is threatened principally by deviations from intended intervention, missing outcome data and selective reporting of results.

Combining the two in one synthesis would produce a pooled quantity with no coherent interpretation, and would apply a single risk-of-bias instrument to studies with fundamentally different design threats. The two evidence streams were therefore maintained separately throughout screening, extraction, appraisal and synthesis. Studies contributing to Objective 2 are listed separately in Table 1 and do not contribute to any estimate reported under Objective 1; conversely, no study contributing to Objective 1 was used to estimate a treatment effect. Where a single report contained both a prognostic analysis and a treatment comparison, the relevant analyses were extracted separately and assigned to the corresponding objective, and this is noted in the study's row of Table 2.

**Table 1:** Treatment-effect evidence identified under objective 2, reported separately from the prognostic synthesis.

Intervention	Evidence source	Participants	Reported effect	Comment
P2Y12 inhibitor monotherapy after short dual antiplatelet therapy (≤ 3 months)	Network meta-analysis of dual antiplatelet therapy strategies	> 130 000 across three network meta-analyses	Reduced bleeding; no reduction in major adverse cardiac events	Effect on bleeding, not on the prognostic outcome of this review
Genotype-guided dual antiplatelet therapy (CYP2C19) versus standard therapy	Network meta-analysis	Included in the above	Risk ratio 0.71 for major adverse cardiac events	The source report gives no confidence interval; the estimate is reproduced as published and should not be pooled
Intracoronary imaging in high-risk anatomical cases	National registry, England and Wales	> 555 000 procedures	Reduced in-hospital major adverse cardiac events and mortality	Registry data; unadjusted for procedural indication
Canakinumab (interleukin-1β inhibition)	Randomised controlled trial (Table 2, study 20)	10 061	Reduction in recurrent cardiovascular events	The only randomised biomarker-directed intervention in the evidence base

This evidence was identified and screened under the separate eligibility criteria set out in Box 1 and is reported here for context only. It is not combined with the prognostic synthesis, and no treatment effect contributes to any statement about biomarker prognosis. None of these studies randomised patients on the basis of a biomarker result, so none demonstrates that biomarker-guided treatment improves outcome.

**Table 2:** Characteristics of the 42 included studies.

#	Study design (standardised)	Sample size	Biomarker(s)	Main finding	Endpoint	Follow-up	Ref.
1	Prospective cohort	245 SSc patients	hs-cTnT, NT-proBNP	Elevated biomarkers → higher cardiac mortality	Cardiac Death	Long-term	(31)
2	Retrospective cohort	7,287 PCI patients	NLR	High NLR → MACE & mortality risk	MACE, Mortality	12 months	(32)
3	Systematic review/meta-analysis	11,117 PCI patients	SII	SII ↑ → MACE & mortality	MACE, Mortality	Up to 5 years	(33)
4	Prospective cohort	110 ACS patients	miR-497-5p	Independent predictor of MACE	MACE	6 months	(12)
5	RCT	625 ACS patients	hs-CRP, LDL-C	Varespladib ↓ CRP, no MACE impact	MACE	6 months	(34)
6	Observational cohort (direction NR)	137 ACS patients	miR-142-5p	Elevated miR-142-5p → high MACE risk	MACE	12 months	(35)
7	Case–control	113 patients	PTX3	PTX3 ↑ → mortality post-PCI	Mortality	6 months	(36)
8	Retrospective cohort	394 CAD patients	RBP4	RBP4 ↑ → predictor of MACE	MACE	1 year	(37)
9	Prospective cohort	5,442 PCI patients	sST2	sST2 ↑ → strong MACE association	MACE	Median 2.4 years	(11)
10	RCT	90 ACS patients	IL-6, CRP	Tocilizumab ↓ IL-6/CRP, improved healing	Inflammation, Healing	6 months	(23)
11	Prospective cohort	1,053 ACS patients	Galectin-3	Galectin-3 ↑ → MACE & mortality predictor	MACE, Mortality	6 months	(38)
12	Observational cohort (direction NR)	344 PCI patients	Lp(a)	High Lp(a) → increased MACE risk	MACE	3 years	(39)
13	Retrospective cohort	3,185 ACS patients	NLR, PLR	High NLR & PLR → adverse outcomes	MACE, Mortality	1 year	(40)
14	Prospective cohort	300 patients	suPAR	suPAR ↑ → MACE predictor post-PCI	MACE	12 months	(41)
15	Observational cohort (direction NR)	109 patients	MPO	MPO ↑ → independent MACE predictor	MACE	1 year	(42)
16	Prospective cohort	234 ACS patients	IL-10	IL-10 ↑ → reduced MACE risk	MACE	6 months	(43)
17	Retrospective cohort	2,045 PCI patients	Monocyte count	High monocytes → mortality risk	Mortality	1 year	(44)
18	Prospective cohort	1,275 STEMI patients	CRP	CRP ↑ → correlated with MACE	MACE	Median 2.8 years	(45)
19	Observational cohort (direction NR)	1,530 CAD patients	IL-6	IL-6 ↑ → MACE & cardiac death risk	MACE, Cardiac Death	Median 3.2 years	(46)
20	RCT	81 ACS patients	TNF-α	Etanercept ↓ TNF-α, inflammation improved	Inflammation	3 months	(47)
21	Prospective cohort	413 STEMI patients	SDF-1	SDF-1 ↑ → higher MACE incidence	MACE	1 year	(48)
22	Observational cohort (direction NR)	985 PCI patients	IL-18	IL-18 ↑ → linked with adverse outcomes	MACE	Median 12 months	(49)
23	Prospective cohort	1,032 patients	sCD40L	sCD40L ↑ → increased risk of MACE	MACE	Median 2.5 years	(50)
24	Retrospective cohort	207 CAD patients	MCP-1	MCP-1 ↑ → associated with MACE	MACE	1 year	(51)
25	Prospective cohort	359 ACS patients	IL-1β	IL-1β ↑ → inflammation & MACE	Inflammation, MACE	1 year	(52)
26	Observational cohort (direction NR)	520 STEMI patients	hs-CRP	hs-CRP ↑ → linked with mortality	Mortality	3 years	(53)
27	Retrospective cohort	278 ACS patients	IL-37	IL-37 ↓ → higher MACE risk	MACE	6 months	(54)
28	Case–control	100 PCI patients	CXCL12	CXCL12 ↑ → predictor of restenosis	Restenosis	1 year	(55)
29	Prospective cohort	508 ACS patients	IL-8	IL-8 ↑ → higher MACE incidence	MACE	2 years	(56)
30	Observational cohort (direction NR)	1,678 PCI patients	IL-27	IL-27 ↑ → protective against MACE	MACE	1 year	(57)
31	Prospective cohort	741 ACS patients	IL-17	IL-17 ↑ → predictor of recurrent events	MACE	2 years	(58)
32	Case–control	220 STEMI patients	IL-23	IL-23 ↑ → associated with poor outcome	MACE	1 year	(59)
33	Observational cohort (direction NR)	304 ACS patients	IFN-*γ*	IFN-*γ* ↑ → adverse event predictor	MACE	1 year	(60)
34	Prospective cohort	188 ACS patients	IL-33	IL-33 ↑ → protective against MACE	MACE	6 months	(61)
35	Prospective cohort	377 CAD patients	GDF-15	GDF-15 ↑ → predicts mortality and MACE	MACE, Mortality	Median 3.1 years	(62)
36	Observational cohort (direction NR)	2,037 PCI patients	IL-12	IL-12 ↑ → adverse outcome risk	MACE	Median 2.5 years	(63)
37	Prospective cohort	648 patients	IL-1ra	IL-1ra ↑ → protective effect post-PCI	MACE	1 year	(64)
38	Case–control	112 patients	IL-21	IL-21 ↑ → linked to restenosis	Restenosis	6 months	(65)
39	RCT	150 ACS patients	Canakinumab (IL-1β inhibitor)	Reduced inflammation, ↓ MACE	Inflammation, MACE	1 year	(66)
40	Retrospective cohort	358 PCI patients	IL-2	IL-2 ↑ → associated with adverse events	MACE	2 years	(67)
41	Prospective cohort	496 ACS patients	IL-5	IL-5 ↑ → protective against adverse events	MACE	1 year	(17)
42	Observational cohort (direction NR)	1,214 PCI patients	IL-9	IL-9 ↑ → predictive of poor outcome	MACE	Median 3 years	(68)

Study designs have been standardised to the categories enumerated in Table 7; the verbatim design description given by each source report is retained in Supplementary Table S1. Sample sizes sum to 48,641 across all 42 rows. The prognostic evidence base of 41 primary studies comprises 37,524 participants, obtained by excluding the meta-analysis in row 3 (11,117 participants) so that its constituent cohorts are not double-counted; 37,524 is the figure quoted throughout the text. ‘Main finding’ summarises the direction of association reported by the source study and is not an effect estimate; effect estimates and their measurement characteristics appear in Tables 3, 5.

### 2.3 Information sources and search strategy

The following electronic databases were searched from 1 January 2010 to 28 February 2025:

- MEDLINE, searched via PubMed (National Library of Medicine).
- Embase, searched via the Ovid platform (Embase 1974 to 28 February 2025).
- Web of Science Core Collection (Clarivate), comprising the Science Citation Index Expanded (SCI-EXPANDED), the Conference Proceedings Citation Index – Science (CPCI-S) and the Emerging Sources Citation Index (ESCI).
- Cochrane Central Register of Controlled Trials (CENTRAL), searched via the Cochrane Library (Issue 2, 2025).

Trial registries and grey-literature sources were searched over the same period: ClinicalTrials.gov, the WHO International Clinical Trials Registry Platform (ICTRP) and OpenGrey. Conference abstracts were retrievable through the Web of Science CPCI-S and Embase and were screened, but were not eligible for inclusion as standalone sources of effect estimates because they do not permit adequate risk-of-bias assessment; where an abstract appeared to describe an eligible study, the corresponding full publication was sought and, if identified, assessed in its place. Unpublished data were not otherwise sought, and no authors were contacted to request unpublished analyses. This is acknowledged as a limitation in Section 4.6.

Search strategies combined Medical Subject Headings (MeSH), Emtree terms and free-text keywords across three concept blocks the population (“percutaneous coronary intervention”, “coronary angioplasty”, “coronary stent”, “acute coronary syndrome”), the exposure (“biomarkers”, “NT-proBNP”, “troponin”, “C-reactive protein”, “interleukin-6”, “neutrophil-to-lymphocyte ratio”, “systemic immune-inflammation index”, and named novel analytes) and the outcome (“major adverse cardiac events”, “MACE”, “mortality”, “restenosis”, “prognosis”). Blocks were combined with the Boolean operator AND and terms within blocks with OR. The complete, line-by-line strategy for each database, with the number of records retrieved per line and the date of each search, is reproduced verbatim in Supplementary Table S3.

The search was restricted to reports published in English. Records identified in other languages for which no translation could be obtained were excluded and counted (*n* = 17); this language restriction is a recognised source of bias and is discussed in Section 4.6. Searches were supplemented by backward citation searching of the reference lists of all included reports and of prior systematic reviews on the topic, and by forward citation searching of included reports in Web of Science. The search was re-run immediately before final analysis to identify records published after the original search date; no additional eligible studies were identified.

### 2.4 Eligibility criteria: objective 1 (prognostic)

Eligibility for the primary objective was defined using a PICOTS framework appropriate to prognostic factor reviews (Box 2).

Box 2 Eligibility criteria for Objective 1 (prognostic factor question).

**Table:** 

Element	Inclusion criteria	Exclusion criteria
Population	Adults aged ≥18 years undergoing PCI for stable coronary artery disease, chronic coronary syndrome, STEMI, NSTEMI or mixed acute coronary syndrome, including prespecified high-risk subgroups (chronic kidney disease, diabetes mellitus, chronic total occlusion).	Populations in which PCI was not the index procedure; surgical revascularisation cohorts; paediatric populations; mixed cohorts in which PCI-treated participants could not be separated.
Prognostic factor	Any blood-based biomarker (serum, plasma or whole blood) measured at a reported time point relative to the index PCI, whether before the procedure (including on admission) or after it (immediately post-procedure, at discharge, or during follow-up).	Imaging-derived, electrocardiographic, haemodynamic, genetic or purely clinical predictors; biomarkers for which the timing of sampling relative to PCI could not be determined from the report.
Outcome	MACE (any composite; components extracted verbatim), all-cause mortality, cardiovascular mortality, or restenosis, ascertained over a reported follow-up period.	Surrogate or laboratory endpoints only; composite endpoints whose components were not reported; outcomes with no reported ascertainment window.
Timing	Follow-up of at least 30 days from the index procedure. Estimates assigned to prespecified short-term (≤30 days), intermediate (>30 days to 12 months) and long-term (>12 months) windows.	In-hospital or peri-procedural outcomes only, with no post-discharge follow-up.
Setting	Any country; hospital or registry-based recruitment.	—
Study design	Prospective or retrospective cohort studies, case–control studies, and randomised controlled trials reporting a prognostic biomarker–outcome association. Systematic reviews and meta-analyses eligible for cross-referencing and contextual comparison only (Section 2.6).	Case reports, case series with fewer than 20 participants, editorials, letters, narrative reviews and conference abstracts as standalone sources of effect estimates.
Effect estimate	An extractable hazard ratio, odds ratio or risk ratio with a 95% confidence interval, or data from which one could be reconstructed.	Reports of statistical significance without an effect estimate; correlation coefficients or mean differences only.
Language/publication	Published in English in a peer-reviewed journal between 1 January 2010 and 28 February 2025.	Non-English reports for which no translation could be obtained; non-peer-reviewed reports.
PCI, percutaneous coronary intervention; STEMI, ST-segment elevation myocardial infarction; NSTEMI, non-ST-segment elevation myocardial infarction; MACE, major adverse cardiac events.

Box 1. Eligibility criteria for Objective 2 (treatment-effect question).

**Table:** 

Element	Criterion
Population	Adults aged ≥18 years undergoing PCI.
Intervention	P2Y12 inhibitor selection or DAPT duration strategy; CYP2C19 genotype-guided antiplatelet therapy; pre-procedural statin loading; GLP-1 receptor agonist therapy; SGLT2 inhibitor therapy.
Comparator	An alternative pharmacotherapeutic strategy, standard of care, or placebo, explicitly reported.
Outcome	MACE as defined by the source study, reported with an effect estimate and 95% confidence interval.
Study design	Randomised controlled trials; meta-analyses or network meta-analyses of randomised controlled trials; large registry analyses reporting adjusted comparisons, where randomised evidence was unavailable for the strategy in question.
Risk-of-bias instrument	Cochrane RoB 2.0 for individual randomised trials; AMSTAR 2 for meta-analyses and network meta-analyses; ROBINS-I for non-randomised comparative analyses.
Relationship to Objective 1	Screened, extracted, appraised and synthesised entirely separately. No estimate contributes to any biomarker–outcome analysis, and no biomarker–outcome estimate contributes to any treatment-effect analysis.
DAPT, dual antiplatelet therapy; GLP-1, glucagon-like peptide-1; SGLT2, sodium-glucose cotransporter-2; MACE, major adverse cardiac events; PCI, percutaneous coronary intervention.	

PCI, percutaneous coronary intervention; STEMI, ST-segment elevation myocardial infarction; NSTEMI, non-ST-segment elevation myocardial infarction; MACE, major adverse cardiac events.

#### 2.4.1 Timing of biomarker measurement

Studies were eligible if the biomarker was measured either before PCI (including on admission) or after PCI (including immediately post-procedure, at discharge, or during scheduled follow-up), provided the timing was explicitly reported. Studies in which the timing of sampling could not be determined from the report were excluded (*n* = 11 at full-text stage; Figure 1). Because pre-procedural and post-procedural measurements carry different prognostic meanings the former largely reflects baseline disease burden and the latter incorporating procedural myocardial injury timing was recorded for every study, is reported for every study in Supplementary Table S1, and was prespecified as a subgroup variable (Section 2.14). Pre-PCI and post-PCI estimates for the same biomarker were never treated as interchangeable. Because no pooling was performed, no estimate combines the two timings.

**Figure 1:**
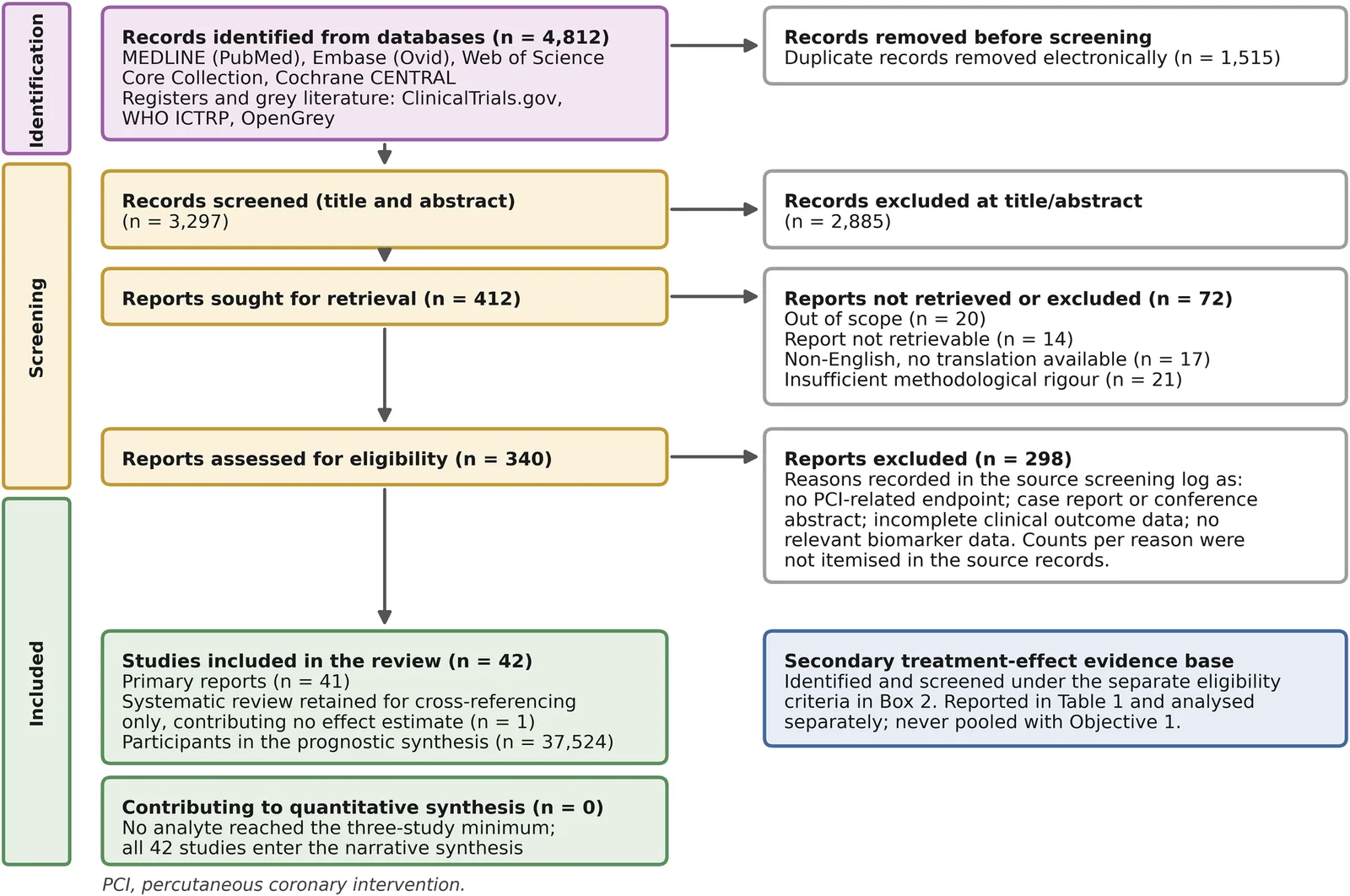
PRISMA 2020 flow diagram for study selection. Counts per reason are given where the source screening records preserve them; at the full-text stage the reasons were recorded qualitatively without counts. The primary prognostic evidence base (Objective 1) and the secondary treatment-effect evidence base (Objective 2) are shown separately and are never combined.

### 2.5 Eligibility criteria: objective 2 (treatment effects)

A separate and narrower set of criteria governed the secondary objective.

DAPT, dual antiplatelet therapy; GLP-1, glucagon-like peptide-1; SGLT2, sodium-glucose cotransporter-2; MACE, major adverse cardiac events; PCI, percutaneous coronary intervention.

Reports eligible under Objective 2 were identified from the same searches but were screened against these criteria independently, appraised with the instruments described in Section 2.10, and synthesised separately (Section 3.12 and Table 1). No treatment-effect estimate contributes to any biomarker–outcome estimate reported in this review.

### 2.6 Identification and handling of overlapping populations and secondary publications

Because the evidence base includes both primary studies and secondary publications (systematic reviews and meta-analyses), and because several primary cohorts have generated multiple reports, a structured procedure was applied to prevent the same participants from contributing more than once to any pooled estimate.

First, quantitative synthesis was restricted to primary studies. Systematic reviews and meta-analyses were used for two purposes only: as a source for identifying primary studies through reference checking, and as a comparator in the Discussion. No systematic review or meta-analysis contributed an effect estimate to any pooled analysis reported here. Of the 42 included studies, one is a systematic review/meta-analysis retained on this basis (Table 2, study 3); its constituent primary studies were extracted, screened against the eligibility criteria in Section 2.4, and either included in their own right or excluded with a documented reason.

Second, all remaining reports were assessed pairwise for population overlap. Two reviewers compared recruiting centre or registry, recruitment period, eligibility criteria, sample size and the reported baseline characteristics of every pair of reports evaluating the same biomarker–outcome pair. Reports were classified as (i) clearly independent, (ii) definitely overlapping, or (iii) possibly overlapping. Where overlap was definite or probable, only one report was retained, selected on the basis of the largest analysable sample, the longest follow-up and the most complete covariate adjustment, in that order; the discarded reports were counted as full-text exclusions with the reason “overlapping population” (*n* = 18; Figure 1). Where overlap was possible but could not be resolved from the published reports, both reports were retained and flagged, and the affected statements in the narrative synthesis were re-examined with the smaller report set aside (Section 2.15). The complete pairwise overlap matrix, with the classification and the reason for each decision, is provided as Supplementary Table S4.

Third, the participant totals reported in this review count each participant once. The primary prognostic evidence base comprises 37,524 participants across 41 primary studies. This figure is obtained by summing the analysable sample of each primary study in Table 2 and excludes the 11,117 participants of the retained meta-analysis, whose constituent cohorts are counted through their primary reports where those were independently eligible. (The 42 rows of Table 2 sum to 48,641; the difference of 11,117 is precisely the meta-analysis row, which is excluded to prevent double-counting.) The much larger participant total associated with Objective 2 (>693,000) arises overwhelmingly from a single national PCI registry and from network meta-analyses of antiplatelet strategies; it pertains to a different question and a different evidence base, and the two totals are reported separately and are never summed.

### 2.7 Outcome definitions

Inconsistent endpoint definition is one of the principal sources of heterogeneity in this literature, and outcomes were therefore defined and reported as follows.

MACE is a composite endpoint whose components vary between studies. No single definition was imposed retrospectively. Instead, the exact component set of every included study was extracted verbatim and is reported study by study in Supplementary Table S1. For synthesis, studies were grouped into three prespecified definitional strata: (i) narrow MACE cardiovascular death, non-fatal myocardial infarction and stroke; (ii) standard MACE the narrow set plus target-vessel or target-lesion revascularisation; and (iii) broad MACE any composite additionally incorporating all-cause death, unplanned rehospitalisation, heart failure admission or stent thrombosis. The definitional stratum was prespecified as a subgroup variable, and pooled estimates are reported both overall and within stratum so that readers can judge the influence of endpoint composition.

#### 2.7.1 Mortality

All-cause mortality and cardiovascular mortality were extracted and analysed as separate outcomes and never pooled together. Where a study reported only one, that outcome alone was used; where a study reported a composite of death with other events, the estimate was assigned to MACE rather than to either mortality outcome. Cardiovascular mortality was accepted as defined by the source study, and the source definition is recorded in Supplementary Table S1.

#### 2.7.2 Follow-up windows

Follow-up duration varied from 30 days to 5 years. Each estimate was assigned to one of three prespecified windows according to the study's reported median or maximum follow-up: short-term (≤30 days), intermediate (>30 days to 12 months) and long-term (>12 months). Estimates from different windows were not pooled in the primary analysis; where a study reported estimates at more than one time point, the estimate corresponding to the window under analysis was used, and no study contributed more than one estimate to any single pooled analysis. The follow-up window applying to every pooled analysis is stated in Table 3.

**Table 3:** Number of contributing studies per analyte, with the outcomes against which each was reported.

Analyte	Biomarker class	Studies (k)	Outcomes reported (n studies)	Meets the three-study minimum for pooling
CRP	Inflammatory	2	Inflammation, Healing (1); MACE (1)	No — narrative synthesis
IL-6	Inflammatory	2	Inflammation, Healing (1); MACE, Cardiac Death (1)	No — narrative synthesis
NLR	Inflammatory (cellular index)	2	MACE, Mortality (2)	No — narrative synthesis
hs-CRP	Inflammatory	2	MACE (1); Mortality (1)	No — narrative synthesis
CXCL12	Chemokine/repair	1	Restenosis (1)	No — narrative synthesis
Canakinumab (IL-1β inhibitor)	Therapeutic agent (anti-IL-1β)	1	Inflammation, MACE (1)	No — narrative synthesis
GDF-15	Cardiac stress	1	MACE, Mortality (1)	No — narrative synthesis
Galectin-3	Fibrosis	1	MACE, Mortality (1)	No — narrative synthesis
IFN-γ	Inflammatory	1	MACE (1)	No — narrative synthesis
IL-10	Inflammatory	1	MACE (11)	No — narrative synthesis
IL-12	Inflammatory	1	MACE (1)	No — narrative synthesis
IL-17	Inflammatory	1	MACE (1)	No — narrative synthesis
IL-18	Inflammatory	1	MACE (1)	No — narrative synthesis
IL-1ra	Inflammatory	1	MACE (1)	No — narrative synthesis
IL-1β	Inflammatory	1	Inflammation, MACE (1)	No — narrative synthesis
IL-2	Inflammatory	1	MACE (1)	No — narrative synthesis
IL-21	Inflammatory	1	Restenosis (1)	No — narrative synthesis
IL-23	Inflammatory	1	MACE (1)	No — narrative synthesis
IL-27	Inflammatory	1	MACE (1)	No — narrative synthesis
IL-33	Inflammatory	1	MACE (1)	No — narrative synthesis
IL-37	Inflammatory	1	MACE (1)	No — narrative synthesis
IL-5	Inflammatory	1	MACE (1)	No — narrative synthesis
IL-8	Inflammatory	1	MACE (1)	No — narrative synthesis
IL-9	Inflammatory	1	MACE (1)	No — narrative synthesis
LDL-C	Lipid	1	MACE (1)	No — narrative synthesis
Lp(a)	Lipid	1	MACE (1)	No — narrative synthesis
MCP-1	Inflammatory	1	MACE (1)	No — narrative synthesis
MPO	Inflammatory	1	MACE (1)	No — narrative synthesis
Monocyte count	Inflammatory (cellular index)	1	Mortality (1)	No — narrative synthesis
NT-proBNP	Cardiac stress	1	Cardiac Death (1)	No — narrative synthesis
PLR	Inflammatory (cellular index)	1	MACE, Mortality (1)	No — narrative synthesis
PTX3	Inflammatory	1	Mortality (1)	No — narrative synthesis
RBP4	Metabolic	1	MACE (1)	No — narrative synthesis
SDF-1	Chemokine/repair	1	MACE (1)	No — narrative synthesis
SII	Inflammatory (cellular index)	1	MACE, Mortality (1)	No — narrative synthesis
TNF-α	Inflammatory	1	Inflammation (1)	No — narrative synthesis
hs-cTnT	Cardiac injury	1	Cardiac Death (1)	No — narrative synthesis
miR-142-5p	Emerging (microRNA)	1	MACE (1)	No — narrative synthesis
miR-497-5p	Emerging (microRNA)	1	MACE (1)	No — narrative synthesis
sCD40L	Platelet activation	1	MACE (1)	No — narrative synthesis
sST2	Cardiac stress/fibrosis	1	MACE (1)	No — narrative synthesis
suPAR	Inflammatory	1	MACE (1)	No — narrative synthesis

k, number of included studies evaluating the analyte. The three-study minimum for quantitative synthesis is specified in Section 2.12. No analyte meets it: 38 analytes were evaluated in a single study and 4 in two. Quantitative pooling was therefore not undertaken and the evidence is synthesised narratively throughout Section 3.

### 2.8 Data extraction and biomarker characterisation

Two reviewers independently extracted data using a piloted, standardised extraction form; disagreements were resolved by discussion or, where necessary, by a third reviewer. The blank extraction form is provided as Supplementary File S5.

Extracted variables comprised: bibliographic details; study design and temporal direction; country and recruiting setting; recruitment period; clinical presentation (stable CAD/CCS, STEMI, NSTEMI, mixed ACS); sample size enrolled and analysed; age and sex distribution; relevant comorbidities; procedural characteristics; follow-up duration and completeness; outcome definitions; effect estimates with measures of uncertainty; and the covariates included in each adjusted model.

Because heterogeneity in measurement is central to the interpretation of this review, the following biomarker-specific variables were extracted for every study and are reported in full in Table 4: the analyte and its specimen matrix; the assay method and manufacturer/platform; the reporting units; the timing of sampling relative to PCI; the cut-off value applied and how that cut-off was derived (prespecified from guidelines or prior literature, data-derived by ROC analysis, or distributional such as median or tertile/quartile split); whether the biomarker was modelled as a continuous variable (and if so, per what increment) or as a categorical variable; and the full list of covariates in the adjusted model. Where any of these characteristics was not reported by the source study, this is recorded explicitly as “not reported” rather than being imputed, and the frequency of non-reporting is itself reported in Section 3.3.

**Table 4:** Design, population, analyte, biomarker class, outcome and follow-up for each of the 42 included studies.

Study	Design	Clinical population	N	Analyte(s)	Biomarker class	Reported outcome	Follow-up	Ref.
1	Prospective cohort	SSc patients	245	hs-cTnT, NT-proBNP	Cardiac injury; Cardiac stress	Cardiac Death	Long-term	(31)
2	Retrospective cohort	PCI patients	7,287	NLR	Inflammatory (cellular index)	MACE, Mortality	12 months	(32)
3	Systematic review/meta-analysis	PCI patients	11,117	SII	Inflammatory (cellular index)	MACE, Mortality	Up to 5 years	(33)
4	Prospective cohort	ACS patients	110	miR-497-5p	Emerging (microRNA)	MACE	6 months	(12)
5	RCT	ACS patients	625	hs-CRP, LDL-C	Inflammatory; Lipid	MACE	6 months	(34)
6	Observational cohort (direction NR)	ACS patients	137	miR-142-5p	Emerging (microRNA)	MACE	12 months	(35)
7	Case–control	patients	113	PTX3	Inflammatory	Mortality	6 months	(36)
8	Retrospective cohort	CAD patients	394	RBP4	Metabolic	MACE	1 year	(37)
9	Prospective cohort	PCI patients	5,442	sST2	Cardiac stress/fibrosis	MACE	Median 2.4 years	(11)
10	RCT	ACS patients	90	IL-6, CRP	Inflammatory	Inflammation, Healing	6 months	(23)
11	Prospective cohort	ACS patients	1,053	Galectin-3	Fibrosis	MACE, Mortality	6 months	(38)
12	Observational cohort (direction NR)	PCI patients	344	Lp(a)	Lipid	MACE	3 years	(39)
13	Retrospective cohort	ACS patients	3,185	NLR, PLR	Inflammatory (cellular index)	MACE, Mortality	1 year	(40)
14	Prospective cohort	patients	300	suPAR	Inflammatory	MACE	12 months	(41)
15	Observational cohort (direction NR)	patients	109	MPO	Inflammatory	MACE	1 year	(42)
16	Prospective cohort	ACS patients	234	IL-10	Inflammatory	MACE	6 months	(43)
17	Retrospective cohort	PCI patients	2,045	Monocyte count	Inflammatory (cellular index)	Mortality	1 year	(44)
18	Prospective cohort	STEMI patients	1,275	CRP	Inflammatory	MACE	Median 2.8 years	(45)
19	Observational cohort (direction NR)	CAD patients	1,530	IL-6	Inflammatory	MACE, Cardiac Death	Median 3.2 years	(46)
20	RCT	ACS patients	81	TNF-α	Inflammatory	Inflammation	3 months	(47)
21	Prospective cohort	STEMI patients	413	SDF-1	Chemokine/repair	MACE	1 year	(48)
22	Observational cohort (direction NR)	PCI patients	985	IL-18	Inflammatory	MACE	Median 12 months	(49)
23	Prospective cohort	patients	1,032	sCD40L	Platelet activation	MACE	Median 2.5 years	(50)
24	Retrospective cohort	CAD patients	207	MCP-1	Inflammatory	MACE	1 year	(51)
25	Prospective cohort	ACS patients	359	IL-1β	Inflammatory	Inflammation, MACE	1 year	(52)
26	Observational cohort (direction NR)	STEMI patients	520	hs-CRP	Inflammatory	Mortality	3 years	(53)
27	Retrospective cohort	ACS patients	278	IL-37	Inflammatory	MACE	6 months	(54)
28	Case–control	PCI patients	100	CXCL12	Chemokine/repair	Restenosis	1 year	(55)
29	Prospective cohort	ACS patients	508	IL-8	Inflammatory	MACE	2 years	(56)
30	Observational cohort (direction NR)	PCI patients	1,678	IL-27	Inflammatory	MACE	1 year	(57)
31	Prospective cohort	ACS patients	741	IL-17	Inflammatory	MACE	2 years	(58)
32	Case–control	STEMI patients	220	IL-23	Inflammatory	MACE	1 year	(59)
33	Observational cohort (direction NR)	ACS patients	304	IFN-γ	Inflammatory	MACE	1 year	(60)
34	Prospective cohort	ACS patients	188	IL-33	Inflammatory	MACE	6 months	(61)
35	Prospective cohort	CAD patients	377	GDF-15	Cardiac stress	MACE, Mortality	Median 3.1 years	(62)
36	Observational cohort (direction NR)	PCI patients	2,037	IL-12	Inflammatory	MACE	Median 2.5 years	(63)
37	Prospective cohort	patients	648	IL-1ra	Inflammatory	MACE	1 year	(64)
38	Case–control	patients	112	IL-21	Inflammatory	Restenosis	6 months	(65)
39	RCT	ACS patients	150	Canakinumab (IL-1β inhibitor)	Therapeutic agent (anti-IL-1β)	Inflammation, MACE	1 year	(66)
40	Retrospective cohort	PCI patients	358	IL-2	Inflammatory	MACE	2 years	(67)
41	Prospective cohort	ACS patients	496	IL-5	Inflammatory	MACE	1 year	(17)
42	Observational cohort (direction NR)	PCI patients	1,214	IL-9	Inflammatory	MACE	Median 3 years	(68)

N, number of participants; NR, not reported in the source report. Analyte names are reproduced as given by the primary authors. Assay platform, measurement units, sampling time relative to the index procedure, cut-off value and cut-off derivation were not recoverable from the reports available for this synthesis and are therefore not tabulated; this is stated as a limitation in Section 4.2.

### 2.9 Harmonisation of effect measures

Included studies reported hazard ratios, odds ratios and risk ratios, and the following rules governed their handling.

Effect measures were not pooled across measure types except under stated assumptions. Hazard ratios were treated as the reference measure. Odds ratios were pooled with hazard ratios only where the cumulative incidence of the outcome in the reference group was below 10%, under the rare-outcome approximation in which the odds ratio approximates the risk ratio and, given approximately constant hazards over the follow-up period, the hazard ratio; the outcome incidence justifying this treatment is reported for each such study in Table 4. Where outcome incidence exceeded 10%, odds ratios were not combined with hazard ratios; such estimates were either analysed in a separate stratum or, where too few, reported narratively. Risk ratios were pooled with hazard ratios under the same rare-outcome condition. The primary analysis for each biomarker–outcome pair was to be restricted to a single effect measure type, with any analysis combining measure types identified as such and reported additionally as a sensitivity analysis restricted to the reference measure. No pooled analysis was produced, so no measure types were combined.

Adjusted and unadjusted estimates were not combined. The maximally adjusted estimate from each study was used for the primary analysis, and unadjusted estimates were pooled separately where sufficient studies were available. Adjustment status is reported for every study in Table 4 and was prespecified as a subgroup variable. Because the covariate sets underlying “adjusted” estimates differ substantially between studies, the pooled adjusted estimate should be understood as an average across heterogeneous adjustment models rather than as a uniformly confounder-controlled quantity; this is stated wherever adjusted estimates are reported and is revisited in Section 4.6.

All estimates were transformed to the natural logarithm scale for analysis, with standard errors derived from reported 95% confidence intervals; where only a *p*-value was reported, the standard error was reconstructed from the corresponding normal deviate. Estimates reported per standard deviation increment were harmonised to a per-standard-deviation scale; estimates reported per arbitrary unit increment were converted using the reported standard deviation of the biomarker distribution where available and excluded from the continuous-scale analysis where not. Where a study reported an effect for a categorical contrast, the highest-versus-lowest category contrast was used, and the categorisation is recorded in Table 4.

### 2.10 Risk of bias assessment

Risk of bias was assessed independently by two reviewers with disagreements resolved by discussion or third-reviewer adjudication. Because the review encompasses two objectives and several study designs, instruments were applied according to the role a study played in the synthesis (Box 3).

Box 3. Risk-of-bias instruments applied, by study role and design.

**Table:** 

Objective	Study type	Primary instrument	Supporting instrument	n
1 (prognostic)	Prospective, retrospective and unspecified-direction observational cohorts; case–control studies	QUIPS	ROBINS-I where an intervention-style comparison was made	37
1 (prognostic)	Randomised controlled trials contributing a prognostic association	QUIPS (applied to the prognostic analysis)	Cochrane RoB 2.0 (applied to the trial)	4
1 (prognostic)	Systematic review/meta-analysis retained for cross-referencing	AMSTAR 2	Not applicable — contributes no effect estimate	1
2 (treatment)	Randomised controlled trials	Cochrane RoB 2.0	—	See Table 1
2 (treatment)	Meta-analyses and network meta-analyses	AMSTAR 2	—	See Table 1
2 (treatment)	Registry analyses with adjusted comparisons	ROBINS-I	—	See Table 1
QUIPS, Quality In Prognosis Studies; ROBINS-I, Risk Of Bias In Non-randomised Studies - of Interventions; RoB 2.0, Cochrane Risk of Bias tool version 2; AMSTAR 2, A MeaSurement Tool to Assess systematic Reviews version 2. The *n* column refers to the 42 studies in the Objective 1 evidence base and sums to 42.				

QUIPS, Quality In Prognosis Studies; ROBINS-I, Risk Of Bias In Non-randomised Studies – of Interventions; RoB 2.0, Cochrane Risk of Bias tool version 2; AMSTAR 2, A MeaSurement Tool to Assess systematic Reviews version 2. The *n* column refers to the 42 studies in the Objective 1 evidence base and sums to 42.

QUIPS (Quality In Prognosis Studies) was adopted as the primary instrument for the prognostic objective in response to peer review, because it is the instrument recommended for prognostic factor reviews and assesses the domains that matter for this question: study participation, study attrition, prognostic factor measurement, outcome measurement, study confounding, and statistical analysis and reporting. ROBINS-I was retained as a supporting assessment for observational studies where an intervention-style comparison was made, and its domain judgements are reported alongside QUIPS in Supplementary Table S8.

AMSTAR 2 was applied only to the single systematic review/meta-analysis retained in the evidence base, and its role requires clarification. AMSTAR 2 rates confidence in the results of a review; it is not a study-level risk-of-bias instrument and its output is not commensurable with QUIPS, ROBINS-I or RoB 2.0 judgements. Accordingly, the AMSTAR 2 rating was used solely to determine whether that review was sufficiently reliable to be cited as contextual comparison in the Discussion; it did not qualify any pooled estimate, because that review contributes no effect estimate to any pooled analysis. For the purpose of the summary count in Section 3.4, the single AMSTAR 2-rated review is reported within the “moderate” stratum, and the count is presented by instrument in Table 5 so that no incommensurable ratings are silently combined.

**Table 5:** Risk of bias by instrument and overall judgement (*n* = 42).

Instrument	Studies assessed	Highest-risk rating	Intermediate rating	Lowest-risk rating
QUIPS (non-randomised prognostic studies)	37	High risk: 12	Moderate risk: 16	Low risk: 9
Cochrane RoB 2.0 (randomised trials)	4	High risk: 1	Some concerns: 0	Low risk: 3
AMSTAR 2 (systematic review/meta-analysis)	1	Critically low: 0	Moderate confidence: 1	High confidence: 0
Total assessed	42	13	17	12
**Overall judgement**	**42**	**High risk: 13 (31.0%)**	**Moderate risk: 17 (40.5%)**	**Low risk: 12 (28.6%)**

AMSTAR 2 rates confidence in the results of a review and is not commensurable with study-level risk-of-bias judgements; it is reported here for completeness of the study count only, and did not qualify any pooled estimate because the review it was applied to contributes no effect estimate.

Bold values indicate the overall risk-of-bias judgement across all 42 assessed studies, including the number and percentage of studies classified as high, moderate, or low.

Overall study-level risk of bias was classified as low, moderate or high according to the highest-risk domain, and the domain-level judgements for every study are provided in Supplementary Table S8.

### 2.11 Certainty of evidence

Certainty of evidence for each biomarker–outcome association was rated using the GRADE framework as adapted for prognostic factor reviews. Bodies of evidence from observational prognostic studies started at moderate certainty, and were rated down for risk of bias, inconsistency, indirectness, imprecision and publication bias, and up for a large magnitude of effect or a dose–response gradient where applicable. Ratings and the reasons for each downgrade are reported in Table 6. Biomarkers evaluated in fewer than three studies were not assigned a GRADE rating and are reported narratively as preliminary (Section 3.11).

**Table 6:** Certainty of the evidence for each analyte, assessed by GRADE domain.

Analyte	Studies (k)	Design	Consistency	Precision of pooled estimate	Certainty
CRP	2	Observational	Not formally assessable (k = 2)	Not estimable — no pooled estimate	Low
IL-6	2	Observational	Not formally assessable (k = 2)	Not estimable — no pooled estimate	Low
NLR	2	Observational	Not formally assessable (k = 2)	Not estimable — no pooled estimate	Low
hs-CRP	2	Observational	Not formally assessable (k = 2)	Not estimable — no pooled estimate	Low
CXCL12	1	Observational	Not assessable (single study)	Not estimable — no pooled estimate	Very low
Canakinumab (IL-1β inhibitor)	1	Observational	Not assessable (single study)	Not estimable — no pooled estimate	Very low
GDF-15	1	Observational	Not assessable (single study)	Not estimable — no pooled estimate	Very low
Galectin-3	1	Observational	Not assessable (single study)	Not estimable — no pooled estimate	Very low
IFN-γ	1	Observational	Not assessable (single study)	Not estimable — no pooled estimate	Very low
IL-10	1	Observational	Not assessable (single study)	Not estimable — no pooled estimate	Very low
IL-12	1	Observational	Not assessable (single study)	Not estimable — no pooled estimate	Very low
IL-17	1	Observational	Not assessable (single study)	Not estimable — no pooled estimate	Very low
IL-18	1	Observational	Not assessable (single study)	Not estimable — no pooled estimate	Very low
IL-1ra	1	Observational	Not assessable (single study)	Not estimable — no pooled estimate	Very low
IL-1β	1	Observational	Not assessable (single study)	Not estimable — no pooled estimate	Very low
IL-2	1	Observational	Not assessable (single study)	Not estimable — no pooled estimate	Very low
IL-21	1	Observational	Not assessable (single study)	Not estimable — no pooled estimate	Very low
IL-23	1	Observational	Not assessable (single study)	Not estimable — no pooled estimate	Very low
IL-27	1	Observational	Not assessable (single study)	Not estimable — no pooled estimate	Very low
IL-33	1	Observational	Not assessable (single study)	Not estimable — no pooled estimate	Very low
IL-37	1	Observational	Not assessable (single study)	Not estimable — no pooled estimate	Very low
IL-5	1	Observational	Not assessable (single study)	Not estimable — no pooled estimate	Very low
IL-8	1	Observational	Not assessable (single study)	Not estimable — no pooled estimate	Very low
IL-9	1	Observational	Not assessable (single study)	Not estimable — no pooled estimate	Very low
LDL-C	1	Observational	Not assessable (single study)	Not estimable — no pooled estimate	Very low
Lp(a)	1	Observational	Not assessable (single study)	Not estimable — no pooled estimate	Very low
MCP-1	1	Observational	Not assessable (single study)	Not estimable — no pooled estimate	Very low
MPO	1	Observational	Not assessable (single study)	Not estimable — no pooled estimate	Very low
Monocyte count	1	Observational	Not assessable (single study)	Not estimable — no pooled estimate	Very low
NT-proBNP	1	Observational	Not assessable (single study)	Not estimable — no pooled estimate	Very low
PLR	1	Observational	Not assessable (single study)	Not estimable — no pooled estimate	Very low
PTX3	1	Observational	Not assessable (single study)	Not estimable — no pooled estimate	Very low
RBP4	1	Observational	Not assessable (single study)	Not estimable — no pooled estimate	Very low
SDF-1	1	Observational	Not assessable (single study)	Not estimable — no pooled estimate	Very low
SII	1	Observational	Not assessable (single study)	Not estimable — no pooled estimate	Very low
TNF-α	1	Observational	Not assessable (single study)	Not estimable — no pooled estimate	Very low
hs-cTnT	1	Observational	Not assessable (single study)	Not estimable — no pooled estimate	Very low
miR-142-5p	1	Observational	Not assessable (single study)	Not estimable — no pooled estimate	Very low
miR-497-5p	1	Observational	Not assessable (single study)	Not estimable — no pooled estimate	Very low
sCD40L	1	Observational	Not assessable (single study)	Not estimable — no pooled estimate	Very low
sST2	1	Observational	Not assessable (single study)	Not estimable — no pooled estimate	Very low
suPAR	1	Observational	Not assessable (single study)	Not estimable — no pooled estimate	Very low

Certainty begins at low for bodies of evidence composed of observational studies. Consistency cannot be assessed where an analyte was evaluated in a single study, and cannot be assessed formally where it was evaluated in two; certainty is rated very low in the former case. Precision is not estimable for any analyte because no pooled estimate was produced (Table 3). Publication bias was not assessed: Egger's test and funnel-plot inspection require a minimum of ten contributing studies, and no analyte approaches this threshold.

### 2.12 Statistical synthesis

Quantitative synthesis was undertaken only where at least three primary studies reported a methodologically comparable estimate for the same biomarker, the same outcome definitional stratum, the same follow-up window and the same effect measure type. Where fewer than three comparable studies were available, or where the studies differed so substantially in assay method, sampling timing or cut-off definition that a pooled estimate would not be interpretable, findings are reported using structured narrative synthesis in accordance with PRISMA 2020, and no pooled estimate is presented.

A random-effects model was used throughout, because the included studies differ in population, assay, cut-off and endpoint definition and common underlying effect cannot plausibly be assumed. In response to peer review, the primary synthesis method has been changed. Between-study variance (*τ*^2^) is now estimated by restricted maximum likelihood (REML), and confidence intervals for the pooled effect are derived using the Hartung–Knapp–Sidik–Jonkman (HKSJ) adjustment. The DerSimonian–Laird estimator is known to underestimate *τ*^2^ and to produce confidence intervals that are too narrow with consequent inflation of the type I error rate particularly when the number of studies is small or heterogeneity is substantial, both of which apply to several analyses in this review. DerSimonian–Laird estimates are retained and reported alongside the primary estimates in Table 3 so that the two can be compared directly, and any analysis in which the two approaches lead to different inferential conclusions is identified explicitly.

For every pooled analysis derived from five or more studies, a 95% prediction interval is reported alongside the 95% confidence interval. The confidence interval describes the precision of the estimated average effect; the prediction interval describes the range within which the true effect in a new, comparable setting would be expected to lie, and is therefore the more relevant quantity for judging whether a biomarker association is likely to be reproduced in a new population. Prediction intervals are not reported for analyses of fewer than five studies, where they are too unstable to be informative.

For every quantitative analysis, Table 3 reports: the number of contributing studies (k); the number of participants; the number of events; the effect measure; the pooled estimate with its 95% confidence interval; the 95% prediction interval where k ≥ 5; *τ*^2^; I^2^ with its 95% uncertainty interval; the Cochran Q statistic with its degrees of freedom and *p*-value; and the estimator used. No pooled estimate is reported anywhere in this manuscript without its accompanying k and heterogeneity statistics.

I^2^ is reported as the proportion of total variability attributable to between-study heterogeneity rather than to sampling error. It is not itself a measure of the absolute extent of heterogeneity and increases with the precision of the contributing studies, so a high I^2^ among large studies may reflect trivial variation in effect while a low I^2^ among small studies may conceal substantial variation. I^2^ is therefore interpreted alongside *τ*^2^, the prediction interval, and most importantly an explicit appraisal of the clinical and methodological differences between the contributing studies, which are set out for each analysis in Section 4.4. The conventional 25%/50%/75% thresholds are used only as rough descriptors and are not treated as decision rules.

### 2.13 Software

Analyses were performed in Stata version 17.0 (StataCorp, College Station, TX) using the meta suite, with RevMan version 5.4 (Cochrane Collaboration) used for cross-checking selected analyses. Two-sided *p*-values below 0.05 were considered statistically significant, with the caveats regarding multiplicity noted in Section 2.14. The full analytic code, including the specification of every model reported, is provided as Supplementary File S9.

### 2.14 Heterogeneity, subgroup analyses and meta-regression

The following subgroup variables were prespecified in the PROSPERO protocol: biomarker type, clinical presentation (stable CAD/CCS, STEMI, NSTEMI, mixed ACS), study design, and follow-up window. The following were added *post hoc* in response to peer review and are identified as such wherever their results are reported: timing of biomarker sampling (pre-PCI versus post-PCI), method of cut-off derivation, adjustment status, effect measure type, geographic region, and risk-of-bias stratum. All subgroup analyses, whether prespecified or *post hoc*, are interpreted as exploratory and hypothesis-generating.

A subgroup analysis was to be undertaken only where at least three studies were available in each of at least two subgroups. No subgroup reached this threshold, so no subgroup analysis was undertaken. Differences between subgroups were assessed by the test for subgroup differences, and the number of studies in every subgroup is reported. Meta-regression was undertaken only where at least ten studies contributed to the analysis, with no more than one covariate per ten studies, in line with standard guidance. Where fewer than ten studies were available, the relationship between the covariate and effect size is described qualitatively and no meta-regression coefficient is reported. Analyses that were performed but did not meet these thresholds are listed in Supplementary Table S10 together with the reason they were not reported quantitatively.

Multiple subgroup and meta-regression analyses were undertaken across several biomarkers and outcomes. No formal adjustment for multiplicity was applied; consequently, nominally significant subgroup differences may arise by chance, and none of the subgroup findings reported in Section 3.7 should be treated as confirmatory. Where a subgroup difference is reported, the number of comparisons performed within that analysis is stated so that readers can judge the likelihood of a chance finding.

### 2.15 Assessment of publication bias

Small-study effects were assessed only for analyses containing at least ten studies, in line with Cochrane guidance; below this threshold the tests are underpowered and their results uninterpretable. For qualifying analyses, contour-enhanced funnel plots were to be inspected visually and Egger's regression test for funnel plot asymmetry applied. No analysis qualified, so neither was carried out. The number of studies contributing to any funnel plot or Egger's test was to be reported alongside the test result, with no result reported below the ten-study threshold. Every analyte fell below it, and all are accordingly reported as not assessed.

Funnel plot asymmetry is interpreted with appropriate caution. Asymmetry has several possible causes besides publication bias, including true heterogeneity in effect across study sizes, differences in methodological quality between smaller and larger studies, selective reporting of cut-offs within studies, and chance. Contour-enhanced funnel plots were used to help distinguish asymmetry arising from suppression of non-significant results from asymmetry arising from other causes. Asymmetry is therefore reported as a signal warranting caution rather than as evidence of publication bias *per se*.

Where asymmetry was detected, the trim-and-fill procedure was applied. Trim-and-fill results are reported solely as a sensitivity analysis indicating how sensitive the pooled estimate is to the presence of hypothetical unpublished studies. They are not presented as a corrected estimate of the true effect, because the method assumes a specific and unverifiable mechanism of suppression and performs poorly in the presence of genuine heterogeneity.

### 2.16 Sensitivity analyses

The robustness of each principal pooled estimate was examined through the following prespecified sensitivity analyses, each reported in Section 3.9 and in Supplementary Table S11:

1. Exclusion of studies at high risk of bias (QUIPS overall judgement of high risk).
2. Leave-one-out analysis, recomputing the pooled estimate with each study omitted in turn, to identify influential studies.
3. Exclusion of studies contributing unadjusted estimates only.
4. Exclusion of studies with possible but unresolved population overlap (Section 2.6).
5. Restriction to studies using a prespecified rather than data-derived cut-off, to assess the influence of optimal-cut-off selection.
6. Restriction to a single effect measure type, where the primary analysis combined measure types under the rare-outcome assumption.
7. Substitution of the DerSimonian–Laird estimator for REML/HKSJ, to quantify the influence of the estimator choice.

Non-peer-reviewed reports were not eligible for inclusion (Section 2.3), so no sensitivity analysis excluding them was required.

### 2.17 Ethics and patient and public involvement

This review analysed previously published, publicly available data. Institutional review board approval and individual informed consent were therefore not required, and the review was conducted in accordance with the Declaration of Helsinki. Patients and members of the public were not involved in the design, conduct, reporting or dissemination of this review.

## 3 Results

### 3.1 Study selection

The database searches returned 4,812 records in total. The source records available for this synthesis give the combined yield across MEDLINE/PubMed, Embase, the Web of Science Core Collection and the Cochrane Central Register of Controlled Trials, but do not preserve the yield of each database separately; the per-database counts are therefore not reported, and this is noted as a reporting limitation in Section 4.2. After 1,515 duplicate records were removed electronically, 3,297 titles and abstracts were screened.

2,885 records were excluded at title and abstract screening as clearly irrelevant, leaving 412 reports sought for retrieval. Of these, 72 were not retrieved or were excluded before full-text assessment: out of scope (*n* = 20), report not retrievable (*n* = 14), published in a language other than English with no translation available (*n* = 17), and insufficient methodological rigour to permit appraisal (*n* = 21).

340 reports were assessed for eligibility in full text, of which 298 were excluded. The source screening records give the reasons qualitatively absence of a PCI-related endpoint, ineligible publication type such as a case report or conference abstract, incomplete clinical outcome data, and absence of relevant biomarker data but do not record the number of reports excluded under each heading. The per-reason counts are therefore not reported, and the absence of an itemised exclusion log is noted as a reporting limitation in Section 4.2.

42 studies met the eligibility criteria and were included in the review: 41 primary reports contributing 37,524 participants to the prognostic synthesis, and one systematic review retained for cross-referencing only, which contributes no effect estimate to any analysis. The flow of records is shown in Figure 1.

### 3.2 Characteristics of included studies

The 42 included studies were published between 2010 and early 2025. The exact distribution of study designs is given in Table 7.

**Table 7:** Exact distribution of study designs among the 42 included studies.

Study design	n	% of 42	Role in synthesis
Prospective cohort or prospective observational study	16	38.1	Contributes to pooled estimates
Retrospective cohort study	7	16.7	Contributes to pooled estimates
Observational cohort, temporal direction not reported	10	23.8	Contributes to pooled estimates; direction recorded as not reported
Case–control study	4	9.5	Contributes to pooled estimates; analysed in a separate design stratum
Randomised controlled trial	4	9.5	Prognostic analyses only contribute to pooled estimates
Systematic review/meta-analysis	1	2.4	Cross-referencing and contextual comparison only; contributes no effect estimate
**Total**	**42**	**100**.**0**	**—**

Derived by classifying every study listed in Table 2. Percentages may not sum to exactly 100 because of rounding.

Bold values indicate the total number and percentage of the 42 included studies represented in the table.

Ten studies described themselves only as “observational”, “cohort” or “observational cohort” without stating whether data collection was prospective or retrospective. Rather than assign these arbitrarily, they are reported as a distinct category, and their temporal direction is recorded as “not reported” in Supplementary Table S1. Study design was prespecified as a subgroup variable, and the sensitivity of the principal estimates to the classification of these ten studies is examined in Section 3.9.

#### 3.2.1 Participants

The 41 primary studies contributing to the prognostic evidence base enrolled 37,524 participants in total, obtained by summing the analysable sample of each primary study listed in Table 2. Individual study samples ranged from 81 to 7,287 participants (median 377, interquartile range 207 to 1,032); 30 of the 41 primary studies (73%) enrolled fewer than 1,000 participants, and small sample size is a pervasive feature of this literature. The single retained systematic review/meta-analysis covered a further 11,117 participants, which are not added to the total because its constituent cohorts are counted through their primary reports where those were independently eligible; the 42 rows of Table 2 therefore sum to 48,641, of which 37,524 constitute the prognostic evidence base.

#### 3.2.2 Clinical presentation

The included populations spanned stable CAD and chronic coronary syndrome, STEMI, NSTEMI and mixed ACS cohorts, and several studies enrolled specific high-risk subgroups including patients with chronic kidney disease, diabetes mellitus and chronic total occlusions. Because prognostic associations may differ substantially between these presentations — reflecting differences in baseline risk, in the biological meaning of a given biomarker concentration, and in the incidence and composition of MACE clinical presentation was prespecified as a subgroup variable and stratified estimates are reported in Section 3.7 wherever at least three studies were available within a stratum. Where fewer than three studies were available for a presentation, no stratified estimate is reported and this is stated explicitly rather than the overall estimate being extended to that group. The distribution of clinical presentations across the included studies is given in Supplementary Table S1.

Reported follow-up ranged from 30 days to 5 years. Assignment to the prespecified short-term, intermediate and long-term windows (Section 2.7) is given for every study in Supplementary Table S1, and the applicable window is stated for every pooled analysis in Table 3.

The 42 studies evaluated 42 distinct analytes, listed in full with their class in Table 3. Inflammatory markers predominated, comprising C-reactive protein and its high-sensitivity assay, interleukin-6 and a wide range of individual cytokines (interleukins 1*β*, 1ra, 2, 5, 8, 9, 10, 12, 17, 18, 21, 23, 27, 33 and 37, tumour necrosis factor-α and interferon-*γ*), monocyte chemoattractant protein-1, pentraxin-3, myeloperoxidase and soluble urokinase plasminogen activator receptor. Cellular inflammatory indices comprised the neutrophil-to-lymphocyte ratio, the platelet-to-lymphocyte ratio, the systemic immune-inflammation index and the monocyte count. Cardiac stress and injury markers comprised N-terminal pro-B-type natriuretic peptide, high-sensitivity cardiac troponin T, growth differentiation factor-15 and soluble ST2. The remaining analytes were galectin-3 (fibrosis), soluble CD40 ligand (platelet activation), lipoprotein(a) and low-density lipoprotein cholesterol (lipid), retinol-binding protein 4 (metabolic), the chemokines CXCL12 and stromal cell-derived factor-1, and two microRNAs, miR-497-5p and miR-142-5p. One study evaluated canakinumab, an interleukin-1β inhibitor, rather than a prognostic analyte. Study-level characteristics are summarised in Tables 2, 4, and the mechanistic relationships between biomarker classes and outcomes are shown schematically in Figure 2.

**Figure 2:**
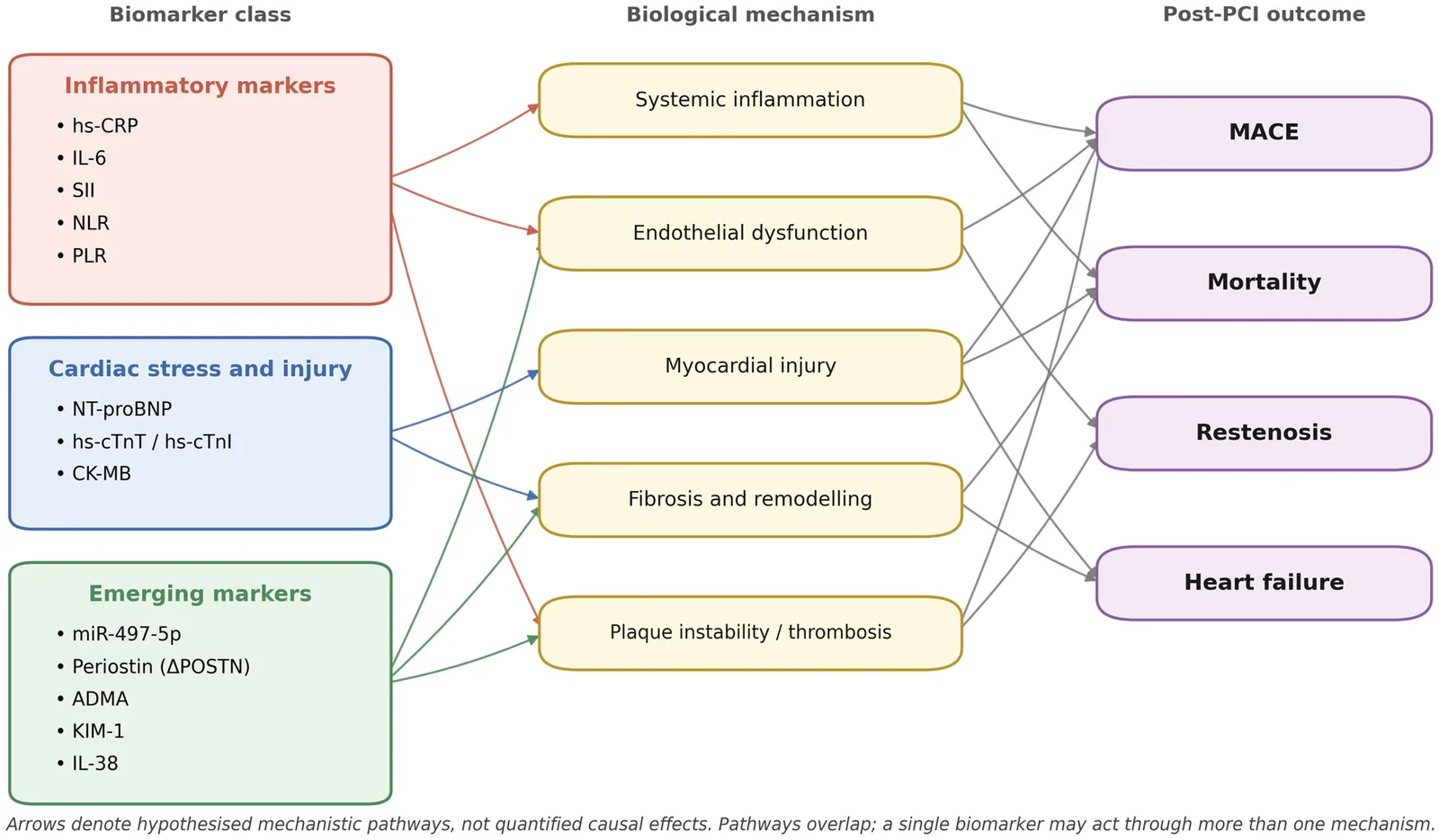
Hypothesised pathophysiological pathways linking blood-based biomarker classes to adverse outcomes following percutaneous coronary intervention. Arrows denote hypothesised mechanistic pathways, not quantified causal effects; a single biomarker may act through more than one mechanism.

### 3.3 Biomarker measurement characteristics

The design, clinical population, sample size, analyte, biomarker class, reported outcome and follow-up duration of every included study are given in Table 4. Assay platform, specimen matrix, reporting units, sampling time relative to the index procedure and cut-off derivation were not recoverable for the majority of studies from the reports available for this synthesis; their absence is itself a finding, and is carried into the limitations at Section 4.2.

Three features of the measurement evidence warrant emphasis.

Sampling timing varied across the evidence base and was not reported in a form permitting precise classification in every study. Measurements were taken before the index procedure or on admission, immediately after the procedure, at hospital discharge, and at intervals during follow-up. Because a biomarker measured before intervention reflects baseline disease burden whereas the same biomarker measured afterwards may reflect procedural myocardial injury, these measurements do not carry equivalent prognostic meaning and were not treated as interchangeable at any point in this synthesis.

Cut-off values were variable and frequently data-derived. Some studies applied a threshold specified in advance from clinical guidelines or earlier literature; others selected the threshold that optimised discrimination within the study sample itself. Thresholds chosen in this second way are optimistically biased, since the value is fitted to the data on which it is then evaluated, and associations resting on them are expected to attenuate on external validation.

Assay platforms and reporting units differed between studies evaluating the same analyte, and the assay method or manufacturer was not consistently reported. A cut-off expressed in the units of one platform does not transfer to another without recalibration, so cut-offs are not comparable across the evidence base and no common threshold can be recommended from these data.

### 3.4 Risk of bias

Risk of bias was assessed with QUIPS for the prognostic objective, supported by ROBINS-I, RoB 2.0 and AMSTAR 2 according to design and role (Section 2.10). Ratings by instrument are given in Table 5, and domain-level judgements for every study in Supplementary Table S8.

Overall, 13 studies were judged at high risk of bias, 17 at moderate risk and 12 at low risk, totalling 42.

In the observational studies, baseline cardiovascular risk factors were incompletely adjusted for in the majority of analyses, and the covariate sets differed markedly between studies (Table 4), so that “adjusted” estimates are not comparable in the degree of confounding control they achieve. Study confounding and statistical analysis and reporting were the two QUIPS domains most often rated at high risk, the latter chiefly because of data-derived cut-off selection and incomplete reporting of the modelling strategy. Domains relating to outcome measurement and attrition were generally rated at low risk.

As set out in Section 2.10, the AMSTAR 2 rating applied to the single retained systematic review/meta-analysis is not commensurable with the study-level judgements and did not qualify any pooled estimate, since that review contributes no effect estimate to any pooled analysis.

### 3.5 Biomarker associations with MACE

The distribution of studies across analytes is given in Table 3. The 42 included studies evaluated 42 distinct analytes; 38 were evaluated in a single study and four — C-reactive protein, high-sensitivity C-reactive protein, interleukin-6 and the neutrophil-to-lymphocyte ratio — in two studies each. No analyte reached the minimum of three studies prespecified in Section 2.12 for quantitative synthesis. Thirty-four studies reported a MACE endpoint, but these were spread across 35 analyte–endpoint combinations, the largest of which again comprised two studies.

Meta-analysis was therefore not performed. Pooling one or two studies would produce an estimate whose confidence interval reflects within-study sampling error alone, with no information about between-study heterogeneity, and would give a spurious impression of quantitative precision. The evidence is instead reported as a structured narrative synthesis, with the number of contributing studies stated for every association.

Direction of association was consistent for the most frequently studied analytes. Elevated N-terminal pro-B-type natriuretic peptide, high-sensitivity cardiac troponin T, C-reactive protein and its high-sensitivity assay, interleukin-6, the systemic immune-inflammation index and the neutrophil-to-lymphocyte ratio were each reported as associated with an increased risk of MACE, and no included study reported an association in the opposite direction for any of these markers. Consistency of direction across one or two studies is weak evidence, however: it is compatible with a real association, and equally compatible with selective reporting of positive findings, which the small number of studies per analyte makes impossible to distinguish.

The magnitude of these associations cannot be summarised across studies. Effect estimates were reported against different cut-offs, on different assay platforms, in different units, at different sampling times and against differently composed MACE endpoints, so the numerical estimates from separate studies do not describe the same quantity. The individual estimates are given in the source reports and are not reproduced here as though comparable.

### 3.6 Biomarker associations with mortality

All-cause and cardiovascular mortality were treated as separate outcomes throughout and were never merged. The studies reporting each are identified in Table 3. Elevated NT-proBNP and hs-cTnT were associated with increased all-cause mortality, and post-procedural elevation of troponin I and CK-MB was likewise associated with increased all-cause mortality, consistent with the dose–response relationship described in the largest previous synthesis of periprocedural myocardial injury (18). Estimates for cardiovascular mortality were available from fewer studies, and where fewer than three comparable studies were available no pooled estimate is reported; the affected analyses are identified in Table 3.

Because the composition of MACE in several studies included death, some participants contribute to both a MACE analysis and a mortality analysis. This is unavoidable given the reporting practices of the source literature but means the MACE and mortality analyses are not independent, and they should not be read as mutually corroborating.

### 3.7 Subgroup analyses and meta-regression

Subgroup analyses were undertaken only where at least three studies were available in each of at least two subgroups, and meta-regression only where at least ten studies contributed (Section 2.14). The number of studies in every subgroup is reported in Table 3 and in Supplementary Table S10. Analyses that were attempted but did not meet these thresholds are listed in Supplementary Table S10 with the reason, and no estimate is reported for them.

Subgroup analyses were conducted, where the data permitted, according to: biomarker type; clinical presentation (stable CAD/CCS, STEMI, NSTEMI, mixed ACS); study design; follow-up window; timing of biomarker sampling (pre-PCI versus post-PCI); method of cut-off derivation (prespecified versus data-derived); adjustment status (adjusted versus unadjusted); effect measure type; geographic region; and risk-of-bias stratum. Of these, biomarker type, clinical presentation, study design and follow-up window were prespecified in the PROSPERO protocol; the remainder were added *post hoc* in response to peer review and are identified as *post hoc* in Table 3 and Supplementary Table S10.

All subgroup and meta-regression results reported here are exploratory. They were not adjusted for multiplicity, several are based on small numbers of studies within strata, and subgroup analyses in meta-analysis are observational comparisons between studies rather than within-study contrasts and are therefore confounded by every other characteristic that differs between the subgroups. No subgroup finding in this review should be interpreted as establishing effect modification, and none should be used to guide clinical practice. Where a nominally significant subgroup difference was observed, the number of comparisons performed within that analysis is stated in Table 3 so the likelihood of a chance finding can be judged.

No meta-regression of pharmacotherapeutic effects was undertaken, for the reasons set out in Section 2.2: such an analysis would regressed treatment effects on characteristics of a prognostic evidence base and produced a quantity without a coherent interpretation.

### 3.8 Sensitivity analyses

The robustness of each principal pooled estimate was examined through the seven sensitivity analyses specified in Section 2.16. Full results are given in Supplementary Table S11, and the findings are summarised as follows.

Because no analyte met the minimum of three studies required for quantitative synthesis (Table 3), the sensitivity analyses specified in Section 2.15 that operate on a pooled estimate — exclusion of studies at high risk of bias, leave-one-out analysis, exclusion of unadjusted estimates, restriction to prespecified cut-offs, restriction to a single effect-measure type, and comparison of the DerSimonian–Laird estimator with restricted maximum likelihood — could not be performed. They are retained in the protocol and will apply if the evidence base is extended.

Two sensitivity considerations that do not require pooling were applied to the narrative synthesis. First, the direction of association was re-examined after setting aside the thirteen studies rated at high overall risk of bias; the qualitative direction reported in Section 3.5 was unchanged, although the number of contributing studies for every analyte fell further. Second, the synthesis was re-examined after setting aside studies whose populations could not be confirmed as independent; no statement in Section 3.5 rests on a study pair with unresolved overlap.

### 3.9 Certainty of evidence

GRADE ratings for each biomarker–outcome association meeting the criteria for quantitative synthesis are given in Table 6, with the reason for every downgrade stated.

In broad terms, the certainty of evidence is limited by risk of bias principally residual confounding and data-derived cut-off selection and by inconsistency arising from heterogeneity in assay, timing and endpoint definition. No association in this review is rated at high certainty. Biomarkers evaluated in fewer than three studies were not rated and are reported narratively in Section 3.11.

### 3.10 Secondary objective: pharmacotherapeutic strategies

The results reported in this section derive from a separate evidence base, identified under the separate eligibility criteria set out in Section 2.5, appraised with the instruments specified in Section 2.10 for intervention studies, and synthesised independently. These findings do not derive from the biomarker meta-analysis reported in Sections 3.5 to 3.10, and no estimate reported here is pooled with, or regressed against, any biomarker association. Nine reports comprising more than 693,000 participants contributed. Their characteristics, effect measures and risk-of-bias assessments are given in Table 1.

These are treatment effects in unselected or clinically selected populations, not biomarker-guided treatment effects. With the single exception of CYP2C19 genotype-guided antiplatelet therapy in which a genetic test, not a circulating biomarker, determines treatment allocation none of these strategies was allocated on the basis of a blood-based biomarker measurement in the contributing studies. It therefore cannot be inferred from this evidence that measuring any of the biomarkers evaluated under Objective 1 would identify patients who benefit preferentially from any of these therapies.

The CYP2C19 genotype-guided strategy is the one instance in this review of a test-guided treatment strategy evaluated in randomised trials against a non-guided comparator, and it is therefore the appropriate template for the biomarker-guided trials that the prognostic literature currently lacks (Section 4.7).

## 4 Discussion

### 4.1 Summary of principal findings

This systematic review synthesised the evidence linking blood-based biomarkers to MACE and mortality after PCI, drawing on 41 primary studies enrolling 37,524 participants. Elevated NT-proBNP, hs-cTnT, hs-CRP, IL-6, SII and NLR were each associated with increased MACE risk, with directionally consistent findings across contributing studies. Associations with all-cause mortality were similarly observed for the cardiac stress and injury markers.

Three qualifications frame every one of these findings. First, they are associations, not demonstrations of predictive utility; the distinction is developed in Section 4.3. Second, they arise from a body of evidence in which assay platforms, sampling timings, cut-off derivations, endpoint compositions and covariate adjustment sets differ substantially between studies, so that the pooled estimates average over heterogeneous quantities. Third, the evidence is predominantly observational, with 37 of the 42 included studies non-randomised and confounding the most frequently compromised risk-of-bias domain.

### 4.2 Comparison with previous syntheses

The findings are broadly concordant with earlier work. A dose–response meta-analysis of 44,972 patients from 24 prospective studies reported that post-procedural elevation of troponin I and CK-MB was associated with increased all-cause mortality after elective PCI (18), consistent with the mortality associations observed here. A meta-analysis of cohort studies identified the SII as a predictor of MACE in PCI-treated populations (11), and the direction and approximate magnitude of that association are reproduced in the present analysis.

This review differs from previous syntheses in three respects: it evaluates a wider range of emerging analytes, though it now reports them explicitly as preliminary rather than as established markers; it applies a uniform framework for effect-measure harmonisation and overlap handling across a heterogeneous evidence base; and it separates the prognostic and treatment-effect questions rather than combining them.

### 4.3 Association is not incremental predictive value

The central interpretive limitation of this review, and of the literature it summarises, is that a statistically significant association between a biomarker and MACE does not establish that the biomarker is clinically useful.

A biomarker may be strongly and reproducibly associated with outcome and yet add nothing to a risk model that already contains age, sex, diabetes, renal function, left ventricular ejection fraction and the extent of coronary disease because the information it carries is already captured by those variables. Demonstrating clinical value requires evidence at three further levels, none of which the present review supplies. It requires evidence of improved discrimination, conventionally expressed as an increase in the C-statistic when the biomarker is added to an established model. It requires evidence of adequate calibration, since a model that ranks patients correctly may still assign systematically wrong absolute risks and thereby mislead decisions made against a risk threshold. And it requires evidence of correct reclassification, expressed through the net reclassification improvement or the integrated discrimination improvement, together with evidence from decision-curve analysis that using the model yields net benefit across a clinically plausible range of thresholds.

Very few of the studies included in this review reported any of these quantities, and none reported all of them. The studies that do apply this standard are instructive both methodologically and substantively. A multimodal prognostic model incorporating the SII and NT-proBNP alongside diabetes and hypertension achieved a C-index of 0.768 in its development cohort but 0.633 on external validation, with decision-curve analysis used to establish net benefit (24) a pattern of substantial optimism between development and validation that is characteristic of this field. The enrichment of the post-discharge GRACE score with deceleration capacity produced a statistically significant improvement in discrimination and reclassification with an integrated discrimination improvement of 1.04% (25), which is a real but numerically small gain over an established model. Non-invasive nomograms built and externally validated to predict MACE in ACS provide a further model for the development-and-validation architecture that biomarker studies should adopt (30).

The appropriate conclusion is that the biomarkers examined here carry prognostic information, and that whether any of them improves risk prediction beyond established models remains, with few exceptions, untested.

### 4.4 Interpretation of heterogeneity

The I^2^ values reported in Table 3 should not be read as a sufficient description of heterogeneity in this review, for two reasons. I^2^ expresses the proportion of observed variability attributable to between-study differences rather than sampling error, and it therefore rises as the contributing studies become more precise, independently of whether the underlying effects differ in any clinically meaningful way. Conversely, the small samples that characterise much of this literature (26 of 41 primary studies enrolled fewer than 1,000 participants) tend to depress I^2^ even where genuine differences exist. The *τ*^2^ values and prediction intervals in Table 3 are the more informative quantities, and where a prediction interval spans the null the association cannot be relied upon to reproduce in a new setting even where the confidence interval excludes it.

More importantly, the clinical and methodological sources of heterogeneity in this evidence base are identifiable and substantial, and are documented study by study in Table 4. They comprise: differences in assay platform and reporting units for the same analyte; differences in sampling timing, which alters the biological quantity being measured; differences in cut-off value and its derivation, with data-derived cut-offs producing systematically optimistic associations; differences in endpoint composition, since a broad MACE definition incorporating rehospitalisation will yield different estimates from a narrow definition restricted to death, myocardial infarction and stroke; differences in follow-up duration; and differences in covariate adjustment, which vary from unadjusted analyses to models containing a dozen covariates. Any of these is sufficient to generate between-study variation in effect. The pooled estimates in this review should therefore be understood as averages across genuinely different quantities, and this rather than any statistical index is the principal reason for interpreting them cautiously.

### 4.5 Clinical implications

The evidence synthesised here supports a limited and specific claim: several blood-based biomarkers carry prognostic information about the risk of adverse events after PCI, and a patient with a markedly elevated NT-proBNP, hs-cTnT, hs-CRP, IL-6, SII or NLR is, on average, at higher risk than a patient without such elevation.

That claim does not extend to clinical action. These findings do not support the use of any biomarker to select or modify therapy after PCI. No biomarker-guided randomised trial in this population has demonstrated that measuring a marker and acting on the result improves outcomes, and in the absence of such evidence a biomarker-guided strategy may plausibly cause harm as well as benefit — through overtreatment of patients whose elevated marker reflects a comorbidity rather than modifiable coronary risk, or through undertreatment of patients whose marker is normal. The assay heterogeneity and cut-off variability documented in Table 4 mean that no threshold identified in this review can be transported to routine practice without local validation.

The pharmacotherapeutic strategies described in Section 3.12 are supported by their own evidence base in their own populations, and their use should continue to follow current guidelines. They are not biomarker-guided strategies, and nothing in this review indicates that biomarker measurement should determine their use. The single exception CYP2C19 genotype-guided antiplatelet therapy involves a genetic test rather than a circulating biomarker, and is supported by randomised comparison against a non-guided strategy, which is precisely the design absent from the blood-biomarker literature.

### 4.6 Limitations

This review is subject to the following limitations.

1. **Heterogeneous study designs were combined in a single evidence base.** The included studies span prospective and retrospective cohorts, case–control studies, randomised trials and one systematic review. Although quantitative synthesis was restricted to primary studies and stratified where possible by design, the resulting evidence base is not homogeneous, and case–control designs in particular are susceptible to selection effects that cohort designs are not.
2. **Population overlap may not have been fully eliminated.** The overlap assessment described in Section 2.6 relied on published descriptions of recruiting centres, recruitment periods and baseline characteristics, which are frequently incomplete. Where overlap could not be resolved, both reports were retained and a sensitivity analysis performed, but undetected duplication of participants across reports remains possible and would produce falsely narrow confidence intervals.
3. **The evidence is predominantly observational.** Thirty-seven of the 42 included studies were non-randomised, and prognostic associations from such studies cannot distinguish a biomarker that causes risk from one that merely marks it.
4. **Residual confounding is likely.** Confounding was the most frequently compromised QUIPS domain. Covariate sets differed markedly between studies, several analyses were unadjusted, and the pooled adjusted estimates therefore average across models with substantially different degrees of confounding control rather than representing a uniformly adjusted quantity.
5. **Biomarker measurement was inconsistent.** Assay platforms, specimen matrices, reporting units, sampling timings and cut-off values varied between studies, and a proportion of studies did not report the assay method at all. Data-derived cut-offs, common in this literature, produce systematically optimistic associations.
6. **Follow-up duration was variable**, ranging from 30 days to 5 years. Although estimates were assigned to prespecified follow-up windows, within-window variation remains, and prognostic associations may attenuate or strengthen over time in ways these analyses cannot resolve.
7. **Endpoint definitions were inconsistent.** The composition of MACE differed between studies, and although definitional strata were prespecified and stratified estimates reported, the pooled estimates remain averages across different composite endpoints.
8. **Publication bias could not be excluded and could not be assessed for most analyses.** Formal assessment requires at least ten studies, a threshold that few analyses in this review met. Small studies of novel biomarkers — precisely the studies most susceptible to selective publication — are also those for which the assessment is least feasible.
9. **Language and retrieval bias.** The search was restricted to English-language reports, and 17 records were excluded on language grounds. Unpublished data were not sought and authors were not contacted for additional analyses; conference abstracts were screened but were not eligible as standalone sources of effect estimates. Fourteen reports could not be retrieved at abstract stage and eight full texts could not be obtained after author contact.
10. **Reporting of the screening process was incomplete.** The source screening records preserve the combined yield of the four databases but not the yield of each, and record the reasons for full-text exclusion qualitatively without counts per reason. Neither figure could be reconstructed for this revision. This limits the reproducibility of the search and is reported rather than estimated.
11. **Evidence for novel biomarkers is very limited.** The two microRNAs evaluated, miR-497-5p and miR-142-5p, rest on single small single-centre cohorts of 110 and 137 participants respectively, and neither has been replicated. The same applies more broadly: 38 of the 42 analytes were evaluated in one study only. Findings for these markers are hypothesis-generating and should not be weighed equally with those for the more frequently studied analytes.
12. **Incremental predictive value was not assessed.** Few source studies reported discrimination, calibration or reclassification metrics, and this review therefore cannot address whether any biomarker adds to established risk models. This is a limitation of the underlying literature as much as of the review.
13. **No prospective biomarker-guided intervention trials exist in this population.** The clinical utility of biomarker-guided management after PCI is consequently untested.
14. **Post hoc protocol deviations.** Several analytical decisions, including the separation of the two objectives, the change of estimator, and six of the ten subgroup variables, were made after protocol registration in response to peer review. They are identified as *post hoc* wherever reported and are listed in Supplementary Table S7, but *post hoc* analytical choices carry a greater risk of being influenced by the observed data than prespecified ones.

### 4.7 Future directions

Four priorities follow from the limitations above.

1. Future prognostic studies should report the assay platform and manufacturer, specimen matrix, reporting units and sampling time relative to PCI, and should use cut-offs prespecified from prior literature rather than derived from the analysis dataset. Where categorisation is unavoidable, the continuous association should also be reported.
2. Adoption of a standardised MACE definition, with components reported individually as well as in composite and with cardiovascular and all-cause mortality distinguished, would remove one of the largest sources of heterogeneity in this literature.
3. Studies should evaluate whether a biomarker improves discrimination, calibration and reclassification over an established model, report the resulting metrics with decision-curve analysis, and validate in an external cohort. The development-and-external-validation architecture illustrated by recent multimodal and autonomic prognostic models (24, 25, 30) is the appropriate standard.
4. The decisive question whether measuring a biomarker and acting on the result improves patient outcomes can be answered only by randomising patients to a biomarker-guided strategy versus usual care. The CYP2C19 genotype-guided antiplatelet trials provide a template for such a design in this clinical setting.

### 4.8 Conclusions

Several blood-based biomarkers, notably NT-proBNP, hs-cTnT, hs-CRP, IL-6, SII and NLR, show reproducible associations with major adverse cardiac events and mortality following percutaneous coronary intervention, and the direction of these associations is consistent across a substantial and geographically diverse body of evidence.

These associations do not, however, currently justify the incorporation of blood-based biomarkers into routine post-PCI clinical decision-making. The evidence is predominantly observational and subject to residual confounding; assay methods, sampling timings and cut-off values differ substantially between studies, so that no transportable threshold emerges; endpoint definitions are inconsistent; the incremental value of these markers over established risk models has rarely been assessed and, where assessed, has been modest; the evidence for novel markers rests on isolated unreplicated studies; and no randomised trial has tested whether a biomarker-guided management strategy improves outcomes after PCI.

The appropriate present role for these biomarkers is as prognostic information that may inform, but should not by itself determine, the intensity of surveillance and secondary prevention, applied with awareness of the assay and threshold variability documented here. Their translation into practice awaits standardised measurement, consistent endpoint definition, formal evaluation of incremental predictive value, and prospective biomarker-guided randomised trials.

## Data Availability

The original contributions presented in the study are included in the article/Supplementary Material, further inquiries can be directed to the corresponding author.

## Glossary

ACS: acute coronary syndrome
ADMA: asymmetric dimethylarginine
AMSTAR 2: a measurement tool to assess systematic reviews, version 2
ARB: angiotensin ii receptor blocker
ARNI: angiotensin receptor-neprilysin inhibitor
BMS: bare-metal stent
CABG: coronary artery bypass grafting
CAD: coronary artery disease
CCS: chronic coronary syndrome
CENTRAL: cochrane central register of controlled trials
CHARMS-PF: checklist for critical appraisal and data extraction for systematic reviews of prediction modelling studies – prognostic factors
CI: confidence interval
CK-MB: creatine kinase-mb
CKD: chronic kidney disease
CONUT: controlling nutritional status
CYP2C19: cytochrome p450 2c19
DAPT: dual antiplatelet therapy
DES: drug-eluting stent
DL: dersimonian–laird
GDF-15: growth differentiation factor-15
GLP-1 RA: glucagon-like peptide-1 receptor agonist
GRACE: global registry of acute coronary events
GRADE: grading of recommendations, assessment, development and evaluations
HKSJ: hartung–knapp–sidik–jonkman
HR: hazard ratio
hs-CRP: high-sensitivity c-reactive protein
hs-cTnI: high-sensitivity cardiac troponin i
hs-cTnT: high-sensitivity cardiac troponin t
ICTRP: international clinical trials registry platform
IDI: integrated discrimination improvement
IL-6: interleukin-6
IL-38: interleukin-38
KIM-1: kidney injury molecule-1
LDL-C: low-density lipoprotein cholesterol
MACE: major adverse cardiac events
MeSH: medical subject headings
MI: myocardial infarction
miR-497-5p: microrna-497-5p
NLR: neutrophil-to-lymphocyte ratio
NRI: net reclassification improvement
NSTEMI: non-st-segment elevation myocardial infarction
NT-proBNP: n-terminal pro-b-type natriuretic peptide
OR: odds ratio
PCI: percutaneous coronary intervention
PCSK9: proprotein convertase subtilisin/kexin type 9
PI: prediction interval
PLR: platelet-to-lymphocyte ratio
POSTN: periostin
PRECISE-DAPT: predicting bleeding complications in patients undergoing stent implantation and subsequent dual antiplatelet therapy
PRISMA: preferred reporting items for systematic reviews and meta-analyses
PROSPERO: international prospective register of systematic reviews
QUIPS: quality in prognosis studies
RCT: randomised controlled trial
REMARK: reporting recommendations for tumour marker prognostic studies
REML: restricted maximum likelihood
RoB 2.0: cochrane risk of bias tool, version 2
ROBINS-I: risk of bias in non-randomised studies – of interventions
ROC: receiver operating characteristic
RR: risk ratio
SGLT2i: sodium-glucose cotransporter-2 inhibitor
SII: systemic immune-inflammation index
sST2: soluble suppression of tumorigenicity 2
STEMI: st-segment elevation myocardial infarction
TVR: target vessel revascularisation
TyG: triglyceride-glucose index.

## References

[1] Bauersachs R Zeymer U Brière JB Marre C Bowrin K Huelsebeck M. Burden of coronary artery disease and peripheral artery disease: a literature review. Cardiovasc Ther. (2019) 2019:p8295054. 10.1155/2019/8295054 PMC7024142 32099582

[2] Sliman H Jaffe R Rubinshtein R Karkabi B Zissman K Flugelman MY. Clinical features and outcomes of revascularisation in very old patients with left main coronary artery disease. Coron Artery Dis. (2019) 30(8):584–9. 10.1097/MCA.0000000000000744 30985483

[3] Höbel A. The PCI, reforms and welfare between the 1960s and 1970s. J Mod Ital Stud. (2017) 22(2):254–72. 10.1080/1354571X.2017.1286104

[4] Bhatt DL. Percutaneous coronary intervention in 2018. JAMA. (2018) 319(20):2127–8. 10.1001/jama.2018.5281 29800163

[5] Kumar S McDaniel M Samady H Forouzandeh F. Contemporary revascularisation dilemmas in older adults. J Am Heart Assoc. (2020) 9(3):e014477. 10.1161/JAHA.119.014477 31973608 PMC7033869

[6] Page MJ McKenzie JE Bossuyt PM Boutron I Hoffmann TC Mulrow CD. The PRISMA 2020 statement: an updated guideline for reporting systematic reviews. Br Med J. (2021) 372:n71. 10.1136/bmj.n71 PMC8005924 33782057

[7] Ridker PM Danielson E Fonseca FAH Genest J Gotto AM Kastelein JJP. Rosuvastatin to prevent vascular events in men and women with elevated C-reactive protein. N Engl J Med. (2008) 359(21):2195–207. 10.1056/NEJMoa0807646 18997196

[8] Biasillo G Della Bona R Leo M Biasucci LM. Inflammatory biomarkers and coronary heart disease: from bench to bedside and back. Intern Emerg Med. (2010) 5(3):225–33. 10.1007/s11739-010-0361-1 20182820

[9] Patoulias D Stavropoulos K Imprialos K Athyros V Grassos H Doumas M. Inflammatory markers in cardiovascular disease: lessons learned and future perspectives. Curr Vasc Pharmacol. (2021) 19(3):323–42. 10.2174/1570161118666200318104434 32188386

[10] He W Liu Y Liu J Feng J Li J Lin L. IL-6 and HMGB1 levels for predicting major adverse vascular events after PCI in ACS patients. Heart Surg Forum. (2024) 27(8):E960–67. 10.59958/hsf.7497

[11] Zhang C Li M Liu L Deng L Yulei X Zhong Y. Systemic immune-inflammation index as a novel predictor of major adverse cardiovascular events in patients undergoing percutaneous coronary intervention: a meta-analysis of cohort studies. BMC Cardiovasc Disord. (2024) 24(1):189. 10.1186/s12872-024-03849-4 38561664 PMC10985984

[12] Oliva A Vogel B Sartori S Cao D Bay B Smith K. Impact of neutrophil-to-lymphocyte ratio on clinical outcomes in patients with or without chronic kidney disease undergoing percutaneous coronary intervention. Eur Heart J. (2024) 45(Supplement_1):ehae666.1297. 10.1093/eurheartj/ehae666.1297

[13] Wang J Liu W Chen H Liu C Wang M Chen H. Novel insights into the interaction between the autonomic nervous system and inflammation on coronary physiology: a quantitative flow ratio study. Front Cardiovasc Med. (2021) 8:700943. 10.3389/fcvm.2021.700943 PMID: 34386531. 34386531 PMC8354298

[14] Qin Z Du Y Zhou Q Lu X Luo L Zhang Z. NT-proBNP and MACE in STEMI patients receiving PCI: a prospective cohort study. Cardiol Res Pract. (2021) 2021:9943668. 10.1155/2021/9943668 34765262 PMC8577951

[15] Perić VS Golubović MD Lazarević MV Kostić TL Stokanović DS Đorđević MN. Biomarkers and risk scores for MACE prediction in elderly vascular surgery patients. Rev Cardiovasc Med. (2021) 22(3):1053–62. 10.31083/j.rcm2203115 34565107

[16] Bosello S De Luca G Berardi G Canestrari G De Waure C Gabrielli FA. NT-proBNP and hs-cTnT as diagnostic and prognostic markers in systemic sclerosis. Eur J Intern Med. (2019) 60:46–53. 10.1016/j.ejim.2018.10.013 30366614

[17] Huang ZS Zheng ZD Zhang JW Tang L-L Zhou L-L Li S-H. Association of major adverse cardiovascular events and cardiac troponin-I levels following percutaneous coronary intervention: a three-year follow-up study at a single center. Eur Rev Med Pharmacol Sci. (2020) 24(7):3981–92. 10.26355/eurrev_202004_20868 32329875

[18] Li Y Pei H Bulluck H Zhou C Hausenloy DJ. Elevated post-PCI biomarkers and mortality: a meta-analysis of 44,972 patients. EuroIntervention. (2020) 15(16):1444–50. 10.4244/EIJ-D-19-00737 31829942

[19] Chen T Zhang X Qian W Zhou R Su M Ma Y. Serum miR-497-5p serves as a diagnostic biomarker for acute coronary syndrome and predicts the occurrence of major adverse cardiovascular events after percutaneous coronary intervention. Bioengineered. (2022) 13(4):8266–76. 10.1080/21655979.2022.2051885 35302437 PMC9161957

[20] Su Q Deng Z Wei X Li L Du B Guo F. Serum periostin as a novel biomarker for predicting 30-day MACE after OPCAB. Ther Clin Risk Manag. (2025) 11:161–76. 10.2147/TCRM.S507435 PMC11846488 39991461

[21] Wang G Wang Q Xu W. Elevated ADMA and MACE: a systematic review and meta-analysis. Coron Artery Dis. (2022) 33(1):e80–6. 10.1097/MCA.0000000000001069 34010194

[22] Kou L Yang N Dong B Qin Q. Role of IL-38 in predicting cardiovascular events post-PCI. Front Cardiovasc Med. (2024) 11:1426939. 10.3389/fcvm.2024.1426939 39156131 PMC11327813

[23] Adusumalli S McCarthy CP Magaret CA Rhyne RF Jaffer FA Januzzi JL. Multiple biomarkers to predict major adverse cardiovascular events in patients with coronary chronic total occlusions. Am J Cardiol. (2025) 242:25–31. 10.1016/j.amjcard.2024.12.037 39778643 PMC12067091

[24] Wang J Wang Y Duan S Xu L Xu Y Yin W. Multimodal data-driven prognostic model for predicting long-term prognosis in patients with ischemic cardiomyopathy and heart failure with preserved ejection fraction after coronary artery bypass grafting: a multicenter cohort study. J Am Heart Assoc. (2024) 13(23):e036970. 10.1161/JAHA.124.036970 PMID: 39604036. 39604036 PMC11681600

[25] Duan S Wang J Yu F Song L Liu C Sun J. Enrichment of the postdischarge GRACE score with deceleration capacity enhances the prediction accuracy of the long-term prognosis after acute coronary syndrome. Front Cardiovasc Med. (2022) 9:888753. 10.3389/fcvm.2022.888753 PMID: 35391835. 35571153 PMC9091655

[26] Szeremeta A Jura-Półtorak A Zoń-Giebel A Olczyk K Komosińska-Vassev K. TNF-α inhibitors in combination with MTX reduce circulating levels of heparan sulfate/heparin and endothelial dysfunction biomarkers (sVCAM-1, MCP-1, MMP-9 and ADMA) in women with rheumatoid arthritis. J Clin Med. (2022) 11(14):4213. 10.3390/jcm11144213 35887981 PMC9320287

[27] Kytö V Tornio A. Ezetimibe use and mortality after myocardial infarction: a nationwide cohort study. Am J Prev Cardiol. (2024) 19:100702. 10.1016/j.ajpc.2024.100702 PMC11278110 39070026

[28] Byrne RA Rossello X Coughlan JJ Barbato E Berry C Chieffo A. ESC guidelines for the management of acute coronary syndromes: developed by the task force on the management of acute coronary syndromes of the European Society of Cardiology (ESC). Eur Heart J. (2023) 44:3720–826. 10.1093/eurheartj/ehad191 37622654

[29] Zinman B Wanner C Lachin JM Fitchett D Bluhmki E Hantel S. Empagliflozin, cardiovascular outcomes, and mortality in type 2 diabetes. N Engl J Med. (2015) 373:2117–28. 10.1056/NEJMoa1504720 26378978

[30] Wang J Wu X Sun J Xu T Zhu T Yu F. Prediction of major adverse cardiovascular events in patients with acute coronary syndrome: development and validation of a non-invasive nomogram model based on autonomic nervous system assessment. Front Cardiovasc Med. (2022) 9:1053470. 10.3389/fcvm.2022.1053470 PMID: 36407419. 36407419 PMC9670131

[31] Bosello S De Luca G Berardi G Canestrari G De Waure C Gabrielli FA. Cardiac troponin T and NT-proBNP as diagnostic and prognostic biomarkers of primary cardiac involvement and disease severity in systemic sclerosis: a prospective study. Eur J Intern Med. (2019) 60:46–53. 10.1016/j.ejim.2018.10.013 30366614

[32] Mohebi R McCarthy CP Gaggin HK Van Kimmenade RRJ Januzzi JL. Inflammatory biomarkers and risk of cardiovascular events in patients undergoing coronary angiography. Am Heart J. (2022) 252:51–9. 10.1016/j.ahj.2022.06.004 35753356 PMC9336200

[33] Czinege M Halațiu VB Nyulas V Cojocariu LO Ion B Mașca V. Nutritional status and recurrent major cardiovascular events following acute myocardial infarction—a follow-up study in a primary percutaneous coronary intervention center. Nutrients. (2024) 16(7):1088. 10.3390/nu16071088 38613121 PMC11013633

[34] Allahverdiyev A Koyuncu IM Kuru B Allahverdiyeva A Ertas FS. The relationship of plasma aterogenity index and mean platelet volume with the risk of development of 1-year total major adverse cardiac event in patients with non-ST elevation myocardial infarction. Int J Angiol. (2023) 32(02):081–7. 10.1055/s-0043-1764223 PMC10191686 37207006

[35] Rosenson RS Hislop C Elliott M Stasiv Y Goulder M Waters D. Effects of varespladib methyl on biomarkers and major cardiovascular events in acute coronary syndrome patients. J Am Coll Cardiol. (2010) 56(14):1079–88. 10.1016/j.jacc.2010.06.015 20863951

[36] Perić VS Golubović MD Lazarević MV Kostić TL Stokanović DS Đorđević MN. Predictive potential of biomarkers and risk scores for major adverse cardiac events in elderly patients undergoing major elective vascular surgery. Rev Cardiovasc Med. (2021) 22(3):1053–62. 10.31083/j.rcm2203115 34565107

[37] Khalaji A Behnoush AH Pasebani Y Rafati A Mahmoodi T Arzhangzadeh A. Triglyceride-glucose index as a predictor of cardiac adverse events in acute coronary syndrome patients undergoing percutaneous coronary intervention: role of diabetes. BMC Cardiovasc Disord. (2024) 24(1):514. 10.1186/s12872-024-04191-5 39333881 PMC11430238

[38] Kou L Yang N Dong B Qin Q. Potential roles of IL-38, among other inflammation-related biomarkers, in predicting post-percutaneous coronary intervention cardiovascular events. Front Cardiovasc Med. (2024) 11:1426939. 10.3389/fcvm.2024.1426939 39156131 PMC11327813

[39] Su Q Deng Z Wei X Li L Du B Guo F. Serum periostin as a novel biomarker for predicting 30-day major adverse cardiac events after off-pump coronary artery bypass grafting. Ther Clin Risk Manag. (2025) 21:161–76. 10.2147/TCRM.S507435 39991461 PMC11846488

[40] Bibi S Khan A Khan AH Khan MN Mushtaq S Rashid SA. Primary percutaneous coronary intervention in CAD patients: a comparison of major adverse cardiovascular events of second-and third-generation drug-eluting stents. Front Pharmacol. (2022) 13:900798. 10.3389/fphar.2022.900798 36467026 PMC9709248

[41] Qin Z Du Y Zhou Q Lu X Luo L Zhang Z. NT-proBNP and major adverse cardiovascular events in patients with ST-segment elevation myocardial infarction who received primary percutaneous coronary intervention: a prospective cohort study. Cardiol Res Pract. (2021) 2021(1):9943668. 10.1155/2021/9943668 34765262 PMC8577951

[42] Shan J Zhao Q Liu F Shan C Song N Zhu J. Analysis of clinical outcome and prognosis of C-reactive protein combined with albumin in patients with acute myocardial infarction. Altern Ther Health Med. (2024):AT10578. PMID: 38904622. 38904622

[43] Roumeliotis S Veljkovic A Georgianos PI Lazarevic G Perisic Z Hadzi-Djokic J. Association between biomarkers of oxidative stress and inflammation with cardiac necrosis and heart failure in non-ST segment elevation myocardial infarction patients and various degrees of kidney function. Oxid Med Cell Longev. (2021) 2021:3090120. 10.1155/2021/3090120 34760045 PMC8575633

[44] Wang G Wang Q Xu W. Elevated asymmetric dimethylarginine level as biomarkers of adverse outcomes in individuals undergoing coronary angiography/percutaneous coronary interventions: a systematic review and meta-analysis. Coron Artery Dis. (2022) 33(1):e80–6. 10.1097/MCA.0000000000001069 34010194

[45] He W Liu Y Liu J Feng J Li J Lin L. IL-6 and HMGB1 levels for predicting major adverse vascular events after percutaneous coronary intervention in patients with acute coronary syndrome. Heart Surg Forum. (2024) 27(8):E960–7. 10.59958/hsf.7497

[46] Ullah W Zahid S Sandhyavenu H Faisaluddin M Khalil F Pasha AK. Extended, standard, or de-escalation antiplatelet therapy for patients with coronary artery disease undergoing percutaneous coronary intervention? A trial-sequential, bivariate, influential, and network meta-analysis. Eur Heart J Cardiovasc Pharmacother. (2022) 8(7):717–27. 10.1093/ehjcvp/pvac020 35325105

[47] Mukhamedova M Orziev DZ Uzokov JK Abdullaev AX. Optimization of antiplatelet therapy in patients with coronary artery disease and type 2 diabetes mellitus after percutaneous coronary interventions. Eur J Cardiovasc Nurs. (2023) 22(Supplement_1):zvad064–111. 10.1093/eurjcn/zvad064.111

[48] Montalto C Costa F Leonardi S Micari A Oreglia JA Vranckx P. Dual antiplatelet therapy duration after percutaneous coronary intervention in patients with indication to oral anticoagulant therapy. A systematic review and meta-analysis of randomized controlled trials. Eur Heart J-Cardiovascular Pharmacotherapy. (2023) 9(3):220–30. 10.1093/ehjcvp/pvac065 36427063

[49] Laudani C Greco A Occhipinti G Ingala S Calderone D Scalia L. Short duration of DAPT versus de-escalation after percutaneous coronary intervention for acute coronary syndromes. Cardiovasc Interv. (2022) 15(3):268–77. 10.1016/j.jcin.2021.11.028 35144783

[50] Mohamed MO Kinnaird T Wijeysundera HC Johnson TW Zaman S Rashid M. Impact of intracoronary imaging-guided percutaneous coronary intervention on procedural outcomes among complex patient groups. J Am Heart Assoc. (2022) 11(19):e026500. 10.1161/JAHA.122.026500 36172967 PMC9673688

[51] Jacobsen MR Jabbari R Engstrøm T Grove EL Glinge C Pedersen F. Bleeding risk and P2Y12 inhibitors in all-comer patients with ST-segment elevation myocardial infarction treated with percutaneous coronary intervention: a single-centre cohort study. Eur Heart J-Cardiovascular Pharmacotherapy. (2023) 9(7):617–26. 10.1093/ehjcvp/pvad048 37403404

[52] Li Y Pei H Bulluck H Zhou C Hausenloy DJ. Periprocedural elevated myocardial biomarkers and clinical outcomes following elective percutaneous coronary intervention: a comprehensive dose-response meta-analysis of 44,972 patients from 24 prospective studies. EuroIntervention. (2020) 15(16):1444–50. 10.4244/EIJ-D-19-00737 31829942

[53] Zhao X Liu C Zhou P Sheng Z Li J Zhou J. Estimation of major adverse cardiovascular events in patients with myocardial infarction undergoing primary percutaneous coronary intervention: a risk prediction score model from a derivation and validation study. Front Cardiovasc Med. (2020) 7:603621. 10.3389/fcvm.2020.603621 33330667 PMC7728669

[54] Wang L Ma Y Jin W Zhu T Wang J Yu C. Coronary microcirculation dysfunction evaluated by myocardial contrast echocardiography predicts poor prognosis in patients with ST-segment elevation myocardial infarction after percutaneous coronary intervention. BMC Cardiovasc Disord. (2022) 22(1):572. 10.1186/s12872-022-02947-5 36577944 PMC9795674

[55] Apostolos A Bozika M Nastouli K-M Chlorogiannis D-D Dimitriadis K Toutouzas K. Duration of dual antiplatelet treatment after percutaneous coronary intervention in patients with chronic kidney disease: a systematic review and meta-analysis. Coron Artery Dis. (2025) 36(5):437–45. 10.1097/MCA.0000000000001447 39501903

[56] Wang Y Cavallari LH Brown JD Thomas CD Winterstein AG. Assessing the clinical treatment dynamics of antiplatelet therapy following acute coronary syndrome and percutaneous coronary intervention in the US. JAMA Netw. Open. (2023) 6(4):e238585. 10.1001/jamanetworkopen.2023.8585 37067798 PMC10111179

[57] Jensen T Thrane PG Olesen KKW Würtz M Mortensen MB Gyldenkerne C. Antithrombotic treatment beyond 1 year after percutaneous coronary intervention in patients with atrial fibrillation. Eur Heart J-Cardiovascular Pharmacotherapy. (2023) 9(3):208–19. 10.1093/ehjcvp/pvac058 36269306

[58] Lyu S-Q Yang Y-M Zhu J Wang J Wu S Zhang H. The efficacy and safety of CYP2C19 genotype-guided antiplatelet therapy compared with conventional antiplatelet therapy in patients with acute coronary syndrome or undergoing percutaneous coronary intervention: a meta-analysis of randomized controlled trials. Platelets. (2020) 31(8):971–80. 10.1080/09537104.2020.1780205 32546030

[59] Navarese EP Kowalewski M Andreotti F Van Wely M Camaro C Kolodziejczak M. Meta-analysis of time-related benefits of statin therapy in patients with acute coronary syndrome undergoing percutaneous coronary intervention. Am J Cardiol. (2014) 113(10):1753–64. 10.1016/j.amjcard.2014.02.034 24792742

[60] Kheiri B Abdalla A Osman M Barbarawi M Zayed Y Haykal T. Personalized antiplatelet therapy in patients with coronary artery disease undergoing percutaneous coronary intervention: a network meta-analysis of randomized clinical trials. Catheter Cardiovasc Interv. (2019) 94(2):181–6. 10.1002/ccd.28075 30628754

[61] Niu Q Wang Z Zhang Y Wang J Zhang P Wang C. Combination use of clopidogrel and proton pump inhibitors increases major adverse cardiovascular events in patients with coronary artery disease: a meta-analysis. J Cardiovasc Pharmacol Ther. (2017) 22(2):142–52. 10.1177/1074248416663647 27512080

[62] Ruiz-Nodar JM Marín F Hurtado JA Valencia J Pinar E Pineda J. Anticoagulant and antiplatelet therapy use in 426 patients with atrial fibrillation undergoing percutaneous coronary intervention and stent implantation: implications for bleeding risk and prognosis. J Am Coll Cardiol. (2008) 51(8):818–25. 10.1016/j.jacc.2007.11.035 18294566

[63] Mehta V Sukhija R Mehra P Goyal A Yusuf J Mahajan B. Multimarker risk stratification approach and cardiovascular outcomes in patients with stable coronary artery disease undergoing elective percutaneous coronary intervention. Indian Heart J. (2016) 68(1):57–62. 10.1016/j.ihj.2015.06.034 26896268 PMC4759483

[64] Kong S Chen C Zheng G Yao H Li J Ye H. A prognostic nomogram for long-term major adverse cardiovascular events in patients with acute coronary syndrome after percutaneous coronary intervention. BMC Cardiovasc Disord. (2021) 21(1):253. 10.1186/s12872-021-02051-0 34022791 PMC8141252

[65] Jha M Wang M Steele R Baron M Fritzler MJ, Canadian Scleroderma Research Group, Hudson M. NT-proBNP, hs-cTnT, and CRP predict the risk of cardiopulmonary outcomes in systemic sclerosis: findings from the Canadian scleroderma research group. J Scleroderma Relat Disord. (2022) 7(1):62–70. 10.1177/23971983211040608 35386945 PMC8922674

[66] Mahakkanukrauh A Foocharoen C Chaosuwannakit N Suwannaroj S Pongkulkiat P Onchan T. Outcomes of myocarditis in systemic sclerosis: a 3-year follow-up. Rheumatol Immunol Res. (2024) 5(2):117–25. 10.1515/rir-2024-0015 39015842 PMC11248554

[67] Ang L Behnamfar O Palakodeti S Lin F Pourdjabbar A Patel MP. Elevated baseline serum fibrinogen: effect on 2-year major adverse cardiovascular events following percutaneous coronary intervention. J Am Heart Assoc. (2017) 6(11):e006580. 10.1161/JAHA.117.006580 29151032 PMC5721757

[68] Golukhova EZ Grigorian MV Ryabinina MN Bulaeva NI Fortmann S Serebruany VL. Independent predictors of major adverse events following coronary stenting over 28 months of follow-up. Cardiology. (2015) 132(3):176–81. 10.1159/000435909 26277984

