# Supplementary material for: Blood-based biomarkers and major adverse cardiac events after percutaneous coronary intervention: a systematic review with narrative synthesis
Source: Front Cardiovasc Med. 2026 Oct 13;13:1817332. doi: 10.3389/fcvm.2026.1817332 (PMC13710230; doi:10.3389/fcvm.2026.1817332)
Supplement: Supplementary file 1 [file Supplementaryfile1.docx]

**Supplementary Material**

**Blood-based biomarkers and major adverse cardiac events after percutaneous coronary intervention: a systematic review with narrative synthesis**

Shyamalendu Tripathy^1*^ and Minesh Patel^2^

^1^School of Pharmacy, Parul University, Vadodara, Gujarat, India; ^2^Parul University, Vadodara, Gujarat, India

*Correspondence: Shyamalendu Tripathy, shyamlendu.tripathy38847@paruluniversity.ac.in

*Frontiers in Cardiovascular Medicine — doi: 10.3389/fcvm.2026.1817332*

## Contents

| **Item** | **Caption** |
| --- | --- |
| Supplementary Table S1 | Characteristics of every included study: design (as reported and standardised) and temporal direction, clinical presentation, biomarker, timing of measurement, verbatim MACE definition, mortality definition, follow-up duration and assigned follow-up window. |
| Supplementary Table S3 | Search strategy for each database and registry, expressed line by line from the prespecified concept blocks. |
| Supplementary Table S4 | Pairwise population-overlap assessment matrix, with the classification and rationale for every decision. |
| Supplementary File S5 | Blank data extraction form as piloted and applied. |
| Supplementary Table S6 | Completed PRISMA 2020 checklist. |
| Supplementary Table S7 | Deviations from the registered PROSPERO protocol (CRD420251076074), with the reason for each. |
| Supplementary Table S8 | Risk-of-bias instrument(s) applied to every included study, with the distribution of judgements (QUIPS, ROBINS-I, RoB 2.0, AMSTAR 2). |
| Supplementary File S9 | Analytic code (Stata do-file) specifying every prespecified model. |
| Supplementary Table S10 | Subgroup and meta-regression analyses attempted, with the number of studies available and the reason any analysis was not reported quantitatively. |
| Supplementary Table S11 | Prespecified sensitivity analyses, with the status and finding of each. |

# Supplementary Table S1. Characteristics of every included study: design (as reported and standardised) and temporal direction, clinical presentation, biomarker, timing of measurement, verbatim MACE definition, mortality definition, follow-up duration and assigned follow-up window

| **#** | **Ref.ᵃ** | **Design as reported by source** | **Standardised design** | **Temporal direction** | **Clinical presentation** | **N** | **Analyte(s)** | **Timing of biomarker measurement** | **Reported outcome(s)** | **MACE definition (verbatim component set; definitional stratum)ᵇ** | **Cardiovascular mortality definition** | **Follow-up (assigned window)** |
| --- | --- | --- | --- | --- | --- | --- | --- | --- | --- | --- | --- | --- |
| 1 | (31) | Prospective Observational | Prospective cohort | Prospective | Systemic sclerosis (presentation NR) | 245 | hs-cTnT, NT-proBNP | Baseline (pre-procedure) | Cardiac death | Not applicable (no MACE endpoint) | Not recorded | Long-term (Long-term (>12 months)) |
| 2 | (32) | Retrospective Cohort | Retrospective cohort | Retrospective | PCI population (presentation NR) | 7,287 | NLR | Admission / pre-PCI | MACE, Mortality | Composite MACE; components not recorded (stratum not assignable) | Not applicable | 12 months (Intermediate (>30 days–12 months)) |
| 3 | (33) | Meta-Analysis | Systematic review/meta-analysis | Not applicable | PCI population (presentation NR) | 11,117 | SII | Baseline | MACE, Mortality | Composite MACE; components not recorded (stratum not assignable) | Not applicable | Up to 5 years (Long-term (>12 months)) |
| 4 | (34) | Prospective Study | Prospective cohort | Prospective | Mixed ACS | 110 | miR-497-5p | Before PCI | MACE | Composite MACE; components not recorded (stratum not assignable) | Not applicable | 6 months (Intermediate (>30 days–12 months)) |
| 5 | (35) | RCT (Phase 2B) | RCT | Prospective (randomised) | Mixed ACS | 625 | hs-CRP, LDL-C | Baseline and follow-up | MACE | Composite MACE; components not recorded (stratum not assignable) | Not applicable | 6 months (Intermediate (>30 days–12 months)) |
| 6 | (36) | Observational | Observational cohort | Not reported | Mixed ACS | 137 | miR-142-5p | Not recorded | MACE | Composite MACE; components not recorded (stratum not assignable) | Not applicable | 12 months (Intermediate (>30 days–12 months)) |
| 7 | (37) | Case-Control | Case–control | Not applicable (case–control) | NR | 113 | PTX3 | Not recorded | Mortality | Not applicable (no MACE endpoint) | Not applicable | 6 months (Intermediate (>30 days–12 months)) |
| 8 | (38) | Retrospective | Retrospective cohort | Retrospective | CAD (stable CAD/CCS not confirmed) | 394 | RBP4 | Not recorded | MACE | Composite MACE; components not recorded (stratum not assignable) | Not applicable | 1 year (Intermediate (>30 days–12 months)) |
| 9 | (39) | Prospective Cohort | Prospective cohort | Prospective | PCI population (presentation NR) | 5,442 | sST2 | Not recorded | MACE | Composite MACE; components not recorded (stratum not assignable) | Not applicable | Median 2.4 years (Long-term (>12 months)) |
| 10 | (40) | RCT | RCT | Prospective (randomised) | Mixed ACS | 90 | IL-6, CRP | Not recorded | Inflammation, Healing | Not applicable (no MACE endpoint) | Not applicable | 6 months (Intermediate (>30 days–12 months)) |
| 11 | (41) | Prospective Study | Prospective cohort | Prospective | Mixed ACS | 1,053 | Galectin-3 | Not recorded | MACE, Mortality | Composite MACE; components not recorded (stratum not assignable) | Not applicable | 6 months (Intermediate (>30 days–12 months)) |
| 12 | (42) | Cohort Study | Observational cohort | Not reported | PCI population (presentation NR) | 344 | Lp(a) | Not recorded | MACE | Composite MACE; components not recorded (stratum not assignable) | Not applicable | 3 years (Long-term (>12 months)) |
| 13 | (43) | Retrospective Study | Retrospective cohort | Retrospective | Mixed ACS | 3,185 | NLR, PLR | Admission | MACE, Mortality | Composite MACE; components not recorded (stratum not assignable) | Not applicable | 1 year (Intermediate (>30 days–12 months)) |
| 14 | (44) | Prospective | Prospective cohort | Prospective | NR | 300 | suPAR | Not recorded | MACE | Composite MACE; components not recorded (stratum not assignable) | Not applicable | 12 months (Intermediate (>30 days–12 months)) |
| 15 | (45) | Observational | Observational cohort | Not reported | NR | 109 | MPO | Not recorded | MACE | Composite MACE; components not recorded (stratum not assignable) | Not applicable | 1 year (Intermediate (>30 days–12 months)) |
| 16 | (46) | Prospective Cohort | Prospective cohort | Prospective | Mixed ACS | 234 | IL-10 | Not recorded | MACE | Composite MACE; components not recorded (stratum not assignable) | Not applicable | 6 months (Intermediate (>30 days–12 months)) |
| 17 | (47) | Retrospective | Retrospective cohort | Retrospective | PCI population (presentation NR) | 2,045 | Monocyte count | Not recorded | Mortality | Not applicable (no MACE endpoint) | Not applicable | 1 year (Intermediate (>30 days–12 months)) |
| 18 | (48) | Prospective Cohort | Prospective cohort | Prospective | STEMI | 1,275 | CRP | Not recorded | MACE | Composite MACE; components not recorded (stratum not assignable) | Not applicable | Median 2.8 years (Long-term (>12 months)) |
| 19 | (49) | Observational Study | Observational cohort | Not reported | CAD (stable CAD/CCS not confirmed) | 1,530 | IL-6 | Baseline | MACE, Cardiac death | Composite MACE; components not recorded (stratum not assignable) | Not recorded | Median 3.2 years (Long-term (>12 months)) |
| 20 | (50) | Randomised Controlled Trial | RCT | Prospective (randomised) | Mixed ACS | 81 | TNF-α | Not recorded | Inflammation | Not applicable (no MACE endpoint) | Not applicable | 3 months (Intermediate (>30 days–12 months)) |
| 21 | (51) | Prospective Study | Prospective cohort | Prospective | STEMI | 413 | SDF-1 | Not recorded | MACE | Composite MACE; components not recorded (stratum not assignable) | Not applicable | 1 year (Intermediate (>30 days–12 months)) |
| 22 | (52) | Observational Cohort | Observational cohort | Not reported | PCI population (presentation NR) | 985 | IL-18 | Not recorded | MACE | Composite MACE; components not recorded (stratum not assignable) | Not applicable | Median 12 months (Intermediate (>30 days–12 months)) |
| 23 | (53) | Prospective Cohort | Prospective cohort | Prospective | NR | 1,032 | sCD40L | Not recorded | MACE | Composite MACE; components not recorded (stratum not assignable) | Not applicable | Median 2.5 years (Long-term (>12 months)) |
| 24 | (54) | Retrospective Study | Retrospective cohort | Retrospective | CAD (stable CAD/CCS not confirmed) | 207 | MCP-1 | Not recorded | MACE | Composite MACE; components not recorded (stratum not assignable) | Not applicable | 1 year (Intermediate (>30 days–12 months)) |
| 25 | (55) | Prospective Study | Prospective cohort | Prospective | Mixed ACS | 359 | IL-1β | Not recorded | Inflammation, MACE | Composite MACE; components not recorded (stratum not assignable) | Not applicable | 1 year (Intermediate (>30 days–12 months)) |
| 26 | (56) | Observational | Observational cohort | Not reported | STEMI | 520 | hs-CRP | Not recorded | Mortality | Not applicable (no MACE endpoint) | Not applicable | 3 years (Long-term (>12 months)) |
| 27 | (57) | Retrospective Cohort | Retrospective cohort | Retrospective | Mixed ACS | 278 | IL-37 | Not recorded | MACE | Composite MACE; components not recorded (stratum not assignable) | Not applicable | 6 months (Intermediate (>30 days–12 months)) |
| 28 | (58) | Case-Control Study | Case–control | Not applicable (case–control) | PCI population (presentation NR) | 100 | CXCL12 | Not recorded | Restenosis | Not applicable (no MACE endpoint) | Not applicable | 1 year (Intermediate (>30 days–12 months)) |
| 29 | (59) | Prospective Study | Prospective cohort | Prospective | Mixed ACS | 508 | IL-8 | Not recorded | MACE | Composite MACE; components not recorded (stratum not assignable) | Not applicable | 2 years (Long-term (>12 months)) |
| 30 | (60) | Cohort Study | Observational cohort | Not reported | PCI population (presentation NR) | 1,678 | IL-27 | Not recorded | MACE | Composite MACE; components not recorded (stratum not assignable) | Not applicable | 1 year (Intermediate (>30 days–12 months)) |
| 31 | (61) | Prospective Cohort | Prospective cohort | Prospective | Mixed ACS | 741 | IL-17 | Not recorded | MACE | Composite MACE; components not recorded (stratum not assignable) | Not applicable | 2 years (Long-term (>12 months)) |
| 32 | (62) | Case-Control | Case–control | Not applicable (case–control) | STEMI | 220 | IL-23 | Not recorded | MACE | Composite MACE; components not recorded (stratum not assignable) | Not applicable | 1 year (Intermediate (>30 days–12 months)) |
| 33 | (63) | Observational | Observational cohort | Not reported | Mixed ACS | 304 | IFN-γ | Not recorded | MACE | Composite MACE; components not recorded (stratum not assignable) | Not applicable | 1 year (Intermediate (>30 days–12 months)) |
| 34 | (64) | Prospective Study | Prospective cohort | Prospective | Mixed ACS | 188 | IL-33 | Not recorded | MACE | Composite MACE; components not recorded (stratum not assignable) | Not applicable | 6 months (Intermediate (>30 days–12 months)) |
| 35 | (65) | Prospective Observational | Prospective cohort | Prospective | CAD (stable CAD/CCS not confirmed) | 377 | GDF-15 | Not recorded | MACE, Mortality | Composite MACE; components not recorded (stratum not assignable) | Not applicable | Median 3.1 years (Long-term (>12 months)) |
| 36 | (66) | Cohort Study | Observational cohort | Not reported | PCI population (presentation NR) | 2,037 | IL-12 | Not recorded | MACE | Composite MACE; components not recorded (stratum not assignable) | Not applicable | Median 2.5 years (Long-term (>12 months)) |
| 37 | (67) | Prospective | Prospective cohort | Prospective | NR | 648 | IL-1ra | Not recorded | MACE | Composite MACE; components not recorded (stratum not assignable) | Not applicable | 1 year (Intermediate (>30 days–12 months)) |
| 38 | (68) | Case-Control | Case–control | Not applicable (case–control) | NR | 112 | IL-21 | Not recorded | Restenosis | Not applicable (no MACE endpoint) | Not applicable | 6 months (Intermediate (>30 days–12 months)) |
| 39 | (69) | RCT | RCT | Prospective (randomised) | Mixed ACS | 150 | Canakinumab (IL-1β inhibitor) | Not recorded | Inflammation, MACE | Composite MACE; components not recorded (stratum not assignable) | Not applicable | 1 year (Intermediate (>30 days–12 months)) |
| 40 | (70) | Retrospective | Retrospective cohort | Retrospective | PCI population (presentation NR) | 358 | IL-2 | Not recorded | MACE | Composite MACE; components not recorded (stratum not assignable) | Not applicable | 2 years (Long-term (>12 months)) |
| 41 | (71) | Prospective Cohort | Prospective cohort | Prospective | Mixed ACS | 496 | IL-5 | Not recorded | MACE | Composite MACE; components not recorded (stratum not assignable) | Not applicable | 1 year (Intermediate (>30 days–12 months)) |
| 42 | (72) | Observational Study | Observational cohort | Not reported | PCI population (presentation NR) | 1,214 | IL-9 | Not recorded | MACE | Composite MACE; components not recorded (stratum not assignable) | Not applicable | Median 3 years (Long-term (>12 months)) |

ᵃReference numbers as printed in Table 2 of the article. ᵇDefinitional strata (Section 2.7): (i) narrow — cardiovascular death, non-fatal myocardial infarction and stroke; (ii) standard — narrow set plus target-vessel or target-lesion revascularisation; (iii) broad — any composite additionally incorporating all-cause death, unplanned rehospitalisation, heart-failure admission or stent thrombosis.

Row 3 is the systematic review/meta-analysis retained for cross-referencing only (Section 2.6); it contributes no effect estimate. Follow-up windows: short-term ≤30 days; intermediate >30 days to 12 months; long-term >12 months. Clinical presentation is given as described by the source report; stable CAD/CCS and NSTEMI populations could not be distinguished where the report described only “CAD” or “PCI” patients.

ACS, acute coronary syndrome; CAD, coronary artery disease; CCS, chronic coronary syndrome; MACE, major adverse cardiac events; NR, not reported in the source report; PCI, percutaneous coronary intervention; RCT, randomised controlled trial; SSc, systemic sclerosis; STEMI, ST-segment elevation myocardial infarction.

Not recorded: item not captured in the review's extraction records. Where the MACE components were not recorded, the definitional stratum could not be assigned, which is one reason no MACE analysis set could be formed (Section 2.12). Sampling time for rows 13 and 19 is as recorded at extraction.

# Supplementary Table S3. Search strategy for each database and registry, expressed line by line from the prespecified concept blocks

## A. Sources searched

| **Source** | **Platform / interface** | **Coverage searched** | **Date search run** | **Records retrieved** |
| --- | --- | --- | --- | --- |
| MEDLINE | PubMed (National Library of Medicine) | 1 January 2010 – 28 February 2025 | Not recorded | 4,812 in total across the four bibliographic databasesᵃ |
| Embase | Ovid (Embase 1974 to 28 February 2025) | 1 January 2010 – 28 February 2025 | Not recorded | See footnote ᵃ |
| Web of Science Core Collection | Clarivate (SCI-EXPANDED; CPCI-S; ESCI) | 1 January 2010 – 28 February 2025 | Not recorded | See footnote ᵃ |
| Cochrane Central Register of Controlled Trials (CENTRAL) | Cochrane Library (Issue 2, 2025) | 1 January 2010 – 28 February 2025 | Not recorded | See footnote ᵃ |
| ClinicalTrials.gov | clinicaltrials.gov | 1 January 2010 – 28 February 2025 | Not recorded | Not recorded |
| WHO International Clinical Trials Registry Platform (ICTRP) | trialsearch.who.int | 1 January 2010 – 28 February 2025 | Not recorded | Not recorded |
| OpenGrey | opengrey.eu | 1 January 2010 – 28 February 2025 | Not recorded | Not recorded |
| Citation searching | Backward (reference lists of included reports and prior reviews); forward (Web of Science) | — | Not recorded | Not recorded |

ᵃThe screening records preserve the combined yield of the four bibliographic databases (4,812 records; 1,515 duplicates removed) but not the yield of each database separately (Section 3.1). The search was re-run immediately before final analysis; no additional eligible studies were identified (Section 2.3).

Not recorded: the run date of each search and the yield of the registers, grey-literature source and citation searching were not retained in the review records.

## B. Concept blocks

| **Block** | **Concept** | **Terms (MeSH/Emtree headings and free-text keywords)** |
| --- | --- | --- |
| 1 | Population | “percutaneous coronary intervention”, “coronary angioplasty”, “coronary stent”, “acute coronary syndrome” |
| 2 | Exposure | “biomarkers”, “NT-proBNP”, “troponin”, “C-reactive protein”, “interleukin-6”, “neutrophil-to-lymphocyte ratio”, “systemic immune-inflammation index”, and named novel analytes |
| 3 | Outcome | “major adverse cardiac events”, “MACE”, “mortality”, “restenosis”, “prognosis” |
| — | Combination and limits | Terms within each block combined with OR; blocks combined with AND (1 AND 2 AND 3). Publication date 1 January 2010 – 28 February 2025; English language. |

## C. Line-by-line strategies

| **Line** | **Search string** |
| --- | --- |
| **MEDLINE (PubMed)** | |
| #1 | "Percutaneous Coronary Intervention"[Mesh] OR "Angioplasty, Balloon, Coronary"[Mesh] OR "Stents"[Mesh] OR "Acute Coronary Syndrome"[Mesh] OR "percutaneous coronary intervention*"[tiab] OR PCI[tiab] OR "coronary angioplast*"[tiab] OR "coronary stent*"[tiab] OR "acute coronary syndrome*"[tiab] |
| #2 | "Biomarkers"[Mesh] OR biomarker*[tiab] OR "Natriuretic Peptide, Brain"[Mesh] OR "NT-proBNP"[tiab] OR "Troponin"[Mesh] OR troponin*[tiab] OR "C-Reactive Protein"[Mesh] OR "C-reactive protein"[tiab] OR CRP[tiab] OR "Interleukin-6"[Mesh] OR interleukin*[tiab] OR "neutrophil-to-lymphocyte ratio"[tiab] OR "neutrophil lymphocyte ratio"[tiab] OR "systemic immune-inflammation index"[tiab] OR microRNA*[tiab] OR periostin[tiab] OR "asymmetric dimethylarginine"[tiab] OR "galectin-3"[tiab] OR sST2[tiab] OR suPAR[tiab] OR "GDF-15"[tiab] |
| #3 | "major adverse cardiac event*"[tiab] OR "major adverse cardiovascular event*"[tiab] OR MACE[tiab] OR "Mortality"[Mesh] OR mortality[tiab] OR death[tiab] OR "Coronary Restenosis"[Mesh] OR restenosis[tiab] OR "Prognosis"[Mesh] OR prognos*[tiab] |
| #4 | #1 AND #2 AND #3 AND ("2010/01/01"[dp] : "2025/02/28"[dp]) AND english[la] |
| **Embase (Ovid)** | |
| #1 | exp percutaneous coronary intervention/ or exp transluminal coronary angioplasty/ or exp coronary stent/ or exp acute coronary syndrome/ or (percutaneous coronary intervention* or PCI or coronary angioplast* or coronary stent* or acute coronary syndrome*).ti,ab. |
| #2 | exp biological marker/ or exp brain natriuretic peptide/ or exp troponin/ or exp C reactive protein/ or exp interleukin 6/ or (biomarker* or NT-proBNP or troponin* or C-reactive protein or CRP or interleukin* or neutrophil-to-lymphocyte ratio or systemic immune-inflammation index or microRNA* or periostin or asymmetric dimethylarginine or galectin-3 or sST2 or suPAR or GDF-15).ti,ab. |
| #3 | exp major adverse cardiac event/ or exp mortality/ or exp restenosis/ or exp prognosis/ or (major adverse cardi* event* or MACE or mortality or death or restenosis or prognos*).ti,ab. |
| #4 | 1 and 2 and 3, limit to (english language and dd=20100101-20250228) |
| **Web of Science Core Collection** | |
| #1 | TS=("percutaneous coronary intervention*" OR PCI OR "coronary angioplast*" OR "coronary stent*" OR "acute coronary syndrome*") |
| #2 | TS=(biomarker* OR "NT-proBNP" OR troponin* OR "C-reactive protein" OR CRP OR interleukin* OR "neutrophil-to-lymphocyte ratio" OR "systemic immune-inflammation index" OR microRNA* OR periostin OR "asymmetric dimethylarginine" OR "galectin-3" OR sST2 OR suPAR OR "GDF-15") |
| #3 | TS=("major adverse cardi* event*" OR MACE OR mortality OR death OR restenosis OR prognos*) |
| #4 | #1 AND #2 AND #3; publication date 2010-01-01 to 2025-02-28; language: English |
| **CENTRAL (Cochrane Library)** | |
| #1 | [mh "Percutaneous Coronary Intervention"] OR [mh "Acute Coronary Syndrome"] OR ("percutaneous coronary intervention*" OR PCI OR "coronary angioplast*" OR "coronary stent*" OR "acute coronary syndrome*"):ti,ab,kw |
| #2 | [mh Biomarkers] OR (biomarker* OR "NT-proBNP" OR troponin* OR "C-reactive protein" OR CRP OR interleukin* OR "neutrophil-to-lymphocyte ratio" OR "systemic immune-inflammation index" OR microRNA* OR periostin OR "asymmetric dimethylarginine"):ti,ab,kw |
| #3 | ("major adverse cardiac event*" OR "major adverse cardiovascular event*" OR MACE OR mortality OR death OR restenosis OR prognos*):ti,ab,kw |
| #4 | #1 AND #2 AND #3, with Cochrane Library publication date from Jan 2010 to Feb 2025, in Trials |
| **ClinicalTrials.gov** | |
| #1 | Condition/disease: "percutaneous coronary intervention" OR "acute coronary syndrome" OR "coronary stent" |
| #2 | Other terms: biomarker OR NT-proBNP OR troponin OR "C-reactive protein" OR interleukin OR "neutrophil-to-lymphocyte ratio" |
| #3 | Outcome measure: MACE OR "major adverse cardiac events" OR mortality OR restenosis |
| #4 | #1 AND #2 AND #3; study start 01/01/2010 to 02/28/2025 |
| **WHO ICTRP** | |
| #1 | Condition: percutaneous coronary intervention OR PCI OR acute coronary syndrome |
| #2 | Title or intervention: biomarker OR NT-proBNP OR troponin OR CRP OR interleukin |
| #3 | Title: MACE OR mortality OR restenosis OR prognosis |
| #4 | #1 AND #2 AND #3; date of registration 01/01/2010 to 28/02/2025 |
| **OpenGrey** | |
| #1 | "percutaneous coronary intervention" OR "coronary angioplasty" OR "coronary stent" |
| #2 | biomarker* OR troponin OR "C-reactive protein" OR interleukin* |
| #3 | MACE OR "major adverse cardiac events" OR mortality |
| #4 | #1 AND #2 AND #3; 2010 to 2025 |

Each strategy expresses the prespecified concept blocks (Section B) in the syntax of the interface concerned, with the date and language limits applied (Section 2.3). The syntax as originally executed and the number of records retrieved per line were not retained in the review records; the combined yield of the four bibliographic databases is given in Section A and Figure 1.

# Supplementary Table S4. Pairwise population-overlap assessment matrix, with the classification and rationale for every decision

## A. Procedure (Section 2.6)

Two reviewers compared recruiting centre or registry, recruitment period, eligibility criteria, sample size and reported baseline characteristics for every pair of reports evaluating the same biomarker–outcome pair. Each pair was classified as (i) clearly independent, (ii) definitely overlapping, or (iii) possibly overlapping. Where overlap was definite or probable, one report was retained, selected on the largest analysable sample, then the longest follow-up, then the most complete covariate adjustment; the other report was excluded at full text with the reason “overlapping population” (n = 18; Figure 1). Where overlap was possible but could not be resolved from the published reports, both reports were retained and flagged, and the affected narrative statements were re-examined with the smaller report set aside (Section 3.8). The systematic review/meta-analysis (Table 2, study 3) was retained for cross-referencing only; its constituent primary studies were screened in their own right.

## B. Included studies evaluating the same analyte

| **Analyte** | **Studies (Table 2 #)** | **Study A (design; population; N; outcome)** | **Study B (design; population; N; outcome)** | **Same biomarker–outcome pair?** | **Classification** | **Decision** | **Rationale** |
| --- | --- | --- | --- | --- | --- | --- | --- |
| NLR | 2 vs 13 | Retrospective cohort; PCI; 7,287; MACE, mortality | Retrospective cohort; ACS; 3,185; MACE, mortality | Yes | Possibly overlapping (pair P1) | Both retained; flagged | Same analyte and outcomes (MACE, mortality) in two retrospective series; recruiting centre/registry and recruitment period could not be established from the published reports, so overlap could not be excluded (Section 2.6). NLR statements in Section 3.5 were re-examined with the smaller report (#13) set aside and did not change (Table S11, analysis 4) |
| CRP | 10 vs 18 | RCT; ACS; 90; inflammation, healing | Prospective cohort; STEMI; 1,275; MACE | No (different outcomes) | Not assessed: different biomarker–outcome pair | Both retained | Overlap assessment applies only to reports of the same biomarker–outcome pair (Section 2.6); the two reports feed different outcome syntheses, so no participant can be counted twice within an analysis |
| IL-6 | 10 vs 19 | RCT; ACS; 90; inflammation, healing | Observational cohort; CAD; 1,530; MACE, cardiac death | No (different outcomes) | Not assessed: different biomarker–outcome pair | Both retained | Overlap assessment applies only to reports of the same biomarker–outcome pair (Section 2.6); the two reports feed different outcome syntheses, so no participant can be counted twice within an analysis |
| hs-CRP | 5 vs 26 | RCT; ACS; 625; MACE | Observational cohort; STEMI; 520; mortality | No (different outcomes) | Not assessed: different biomarker–outcome pair | Both retained | Overlap assessment applies only to reports of the same biomarker–outcome pair (Section 2.6); the two reports feed different outcome syntheses, so no participant can be counted twice within an analysis |
| SII | 3 (meta-analysis) | Systematic review/meta-analysis; PCI; 11,117; MACE, mortality | Constituent primary cohorts | — | Handled by rule (Section 2.6) | Retained for cross-referencing only; contributes no effect estimate | Prevents double-counting of constituent cohorts |

## C. Reports excluded at full text as “overlapping population” (n = 18) and pairs retained as possibly overlapping

| **#** | **Excluded report (citation)** | **Retained report (Table 2 #)** | **Biomarker–outcome pair** | **Shared centre/registry and recruitment period** | **Classification** | **Decision** | **Reason for retaining the chosen report** |
| --- | --- | --- | --- | --- | --- | --- | --- |
| 1–18 | Individual citations not recorded | Not recorded | Not recorded | Not recorded | Definitely/probably overlapping | Excluded (n = 18) | Retention rule applied: largest analysable sample, then longest follow-up, then most complete covariate adjustment (Section 2.6) |
| **Pairs retained and flagged as possibly overlapping** | | | | | | | |
| P1 | None (both reports retained) | 2 and 13 | NLR – MACE, mortality | Not determinable from the published reports | Possibly overlapping | Both retained; flagged | Overlap possible but unresolved; both retained and flagged (Section 2.6). Statements re-examined with #13 (smaller sample) set aside; unchanged |

ACS, acute coronary syndrome; CAD, coronary artery disease; CRP, C-reactive protein; hs-CRP, high-sensitivity CRP; IL-6, interleukin-6; MACE, major adverse cardiac events; NLR, neutrophil-to-lymphocyte ratio; PCI, percutaneous coronary intervention; RCT, randomised controlled trial; SII, systemic immune-inflammation index; STEMI, ST-segment elevation myocardial infarction.

The number of reports excluded as overlapping populations (n = 18; Figure 1) was recorded, but the individual citations and the matched retained reports were not retained in the screening records. Studies 2 and 13 are the only included studies that share a biomarker–outcome pair and are therefore the pair retained and flagged as possibly overlapping (P1).

# Supplementary File S5. Blank data extraction form as piloted and applied

Completed independently by two reviewers for every report; disagreements resolved by discussion or by a third reviewer (Section 2.8). Record “NR” where an item is not reported by the source; do not impute.

## A. Record details

| **Item** | **Field** | **Entry** |
| --- | --- | --- |
| A1 | Study ID (review) / Table 2 row |  |
| A2 | Reviewer initials; date of extraction |  |
| A3 | First author, year, journal, DOI |  |
| A4 | Related reports from the same cohort or registry (for overlap assessment, Section 2.6) |  |
| A5 | Objective served | ☐ Objective 1 (prognostic) ☐ Objective 2 (treatment effect) ☐ Both — extract separately |

## B. Design and setting

| **Item** | **Field** | **Entry** |
| --- | --- | --- |
| B1 | Design as described by the source report (verbatim) |  |
| B2 | Standardised design | ☐ Prospective cohort ☐ Retrospective cohort ☐ Observational cohort, direction NR ☐ Case–control ☐ RCT ☐ Systematic review/meta-analysis |
| B3 | Temporal direction | ☐ Prospective ☐ Retrospective ☐ NR |
| B4 | Country; recruiting setting (single/multicentre; hospital/registry) |  |
| B5 | Recruitment period |  |

## C. Participants

| **Item** | **Field** | **Entry** |
| --- | --- | --- |
| C1 | Clinical presentation | ☐ Stable CAD/CCS ☐ STEMI ☐ NSTEMI ☐ Mixed ACS ☐ Other/NR |
| C2 | Prespecified high-risk subgroup | ☐ CKD ☐ Diabetes mellitus ☐ Chronic total occlusion ☐ None |
| C3 | Key eligibility criteria |  |
| C4 | Sample size enrolled; sample size analysed |  |
| C5 | Age (mean/median); % female |  |
| C6 | Relevant comorbidities |  |
| C7 | Procedural characteristics (access, stent type, lesion complexity) |  |
| C8 | Follow-up duration (median/maximum) and completeness (% lost) |  |

## D. Biomarker characterisation

| **Item** | **Field** | **Entry** |
| --- | --- | --- |
| D1 | Analyte(s) |  |
| D2 | Specimen matrix | ☐ Serum ☐ Plasma ☐ Whole blood ☐ NR |
| D3 | Assay method; manufacturer/platform |  |
| D4 | Reporting units |  |
| D5 | Timing of sampling relative to PCI | ☐ Pre-PCI / admission ☐ Immediately post-PCI ☐ Discharge ☐ Scheduled follow-up ☐ Not determinable (exclude) |
| D6 | Cut-off value |  |
| D7 | Cut-off derivation | ☐ Prespecified (guideline/prior literature) ☐ Data-derived (e.g. ROC) ☐ Distributional (median/tertile/quartile) ☐ NR |
| D8 | Modelling | ☐ Continuous (per unit / per SD: ____) ☐ Categorical (categories: ____) |
| D9 | Contrast used (e.g. highest vs lowest category) |  |

## E. Outcomes

| **Item** | **Field** | **Entry** |
| --- | --- | --- |
| E1 | MACE: components as defined by the source (verbatim) | ☐ CV death ☐ All-cause death ☐ MI ☐ Stroke ☐ TVR/TLR ☐ Rehospitalisation ☐ HF admission ☐ Stent thrombosis ☐ Other: ____ |
| E2 | MACE definitional stratum | ☐ Narrow ☐ Standard ☐ Broad |
| E3 | All-cause mortality reported? | ☐ Yes ☐ No |
| E4 | Cardiovascular mortality reported? Source definition |  |
| E5 | Restenosis or other outcome; definition |  |
| E6 | Outcome ascertainment window; assigned follow-up window | ☐ Short-term (≤30 d) ☐ Intermediate (>30 d–12 mo) ☐ Long-term (>12 mo) |

## F. Effect estimates (one line per biomarker–outcome–time point)

| **Item** | **Field** | **Entry** |
| --- | --- | --- |
| F1 | Outcome and time point |  |
| F2 | Effect measure | ☐ HR ☐ OR ☐ RR |
| F3 | Estimate; 95% CI; p-value |  |
| F4 | Adjustment status | ☐ Adjusted ☐ Unadjusted |
| F5 | Covariates in the adjusted model (full list) |  |
| F6 | Events / participants; cumulative incidence in the reference group (for the rare-outcome rule, Section 2.9) |  |
| F7 | Discrimination, calibration or reclassification metrics (C-statistic, NRI, IDI, calibration), if reported |  |

## G. Objective 2 (treatment-effect reports only)

| **Item** | **Field** | **Entry** |
| --- | --- | --- |
| G1 | Intervention strategy; comparator |  |
| G2 | Design | ☐ RCT ☐ Meta-analysis/NMA of RCTs ☐ Registry with adjusted comparison |
| G3 | MACE definition; effect measure; estimate; 95% CI |  |

## H. Appraisal and reconciliation

| **Item** | **Field** | **Entry** |
| --- | --- | --- |
| H1 | Risk-of-bias instrument(s) assigned (Box 3) | ☐ QUIPS ☐ ROBINS-I ☐ RoB 2.0 ☐ AMSTAR 2 |
| H2 | Discrepancies between reviewers and how resolved |  |
| H3 | Notes |  |

ACS, acute coronary syndrome; CAD, coronary artery disease; CCS, chronic coronary syndrome; CI, confidence interval; CKD, chronic kidney disease; CV, cardiovascular; HF, heart failure; HR, hazard ratio; IDI, integrated discrimination improvement; MI, myocardial infarction; NMA, network meta-analysis; NRI, net reclassification improvement; NSTEMI, non-ST-segment elevation MI; OR, odds ratio; RCT, randomised controlled trial; ROC, receiver operating characteristic; RR, risk ratio; SD, standard deviation; STEMI, ST-segment elevation MI; TLR/TVR, target-lesion/target-vessel revascularisation.

# Supplementary Table S6. Completed PRISMA 2020 checklist

| **Section and topic** | **Item #** | **Checklist item** | **Location where item is reported** |
| --- | --- | --- | --- |
| **TITLE** | | | |
| Title | 1 | Identify the report as a systematic review. | Title |
| **ABSTRACT** | | | |
| Abstract | 2 | See the PRISMA 2020 for Abstracts checklist. | Abstract |
| **INTRODUCTION** | | | |
| Rationale | 3 | Describe the rationale for the review in the context of existing knowledge. | Sections 1–1.5 |
| Objectives | 4 | Provide an explicit statement of the objective(s) or question(s) the review addresses. | Section 1.6 |
| **METHODS** | | | |
| Eligibility criteria | 5 | Specify the inclusion and exclusion criteria for the review and how studies were grouped for the syntheses. | Sections 2.4, 2.5; Boxes 1, 2 |
| Information sources | 6 | Specify all databases, registers, websites, organisations, reference lists and other sources searched or consulted to identify studies. Specify the date when each source was last searched or consulted. | Section 2.3; Supplementary Table S3 |
| Search strategy | 7 | Present the full search strategies for all databases, registers and websites, including any filters and limits used. | Section 2.3; Supplementary Table S3 |
| Selection process | 8 | Specify the methods used to decide whether a study met the inclusion criteria of the review, including how many reviewers screened each record and each report retrieved, whether they worked independently, and if applicable, details of automation tools used in the process. | Section 2.3 — the revised Methods do not state how many reviewers screened records or whether they worked independently |
| Data collection process | 9 | Specify the methods used to collect data from reports, including how many reviewers collected data from each report, whether they worked independently, any processes for obtaining or confirming data from study investigators, and if applicable, details of automation tools used in the process. | Section 2.8; Supplementary File S5 |
| Data items | 10a | List and define all outcomes for which data were sought. Specify whether all results that were compatible with each outcome domain in each study were sought (e.g. for all measures, time points, analyses), and if not, the methods used to decide which results to collect. | Sections 2.7, 2.7.1, 2.7.2 |
|  | 10b | List and define all other variables for which data were sought (e.g. participant and intervention characteristics, funding sources). Describe any assumptions made about any missing or unclear information. | Section 2.8 |
| Study risk of bias assessment | 11 | Specify the methods used to assess risk of bias in the included studies, including details of the tool(s) used, how many reviewers assessed each study and whether they worked independently, and if applicable, details of automation tools used in the process. | Section 2.10; Box 3 |
| Effect measures | 12 | Specify for each outcome the effect measure(s) (e.g. risk ratio, mean difference) used in the synthesis or presentation of results. | Sections 1.6.1, 2.9 |
| Synthesis methods | 13a | Describe the processes used to decide which studies were eligible for each synthesis (e.g. tabulating the study intervention characteristics and comparing against the planned groups for each synthesis (item #5)). | Section 2.12; Table 3 |
|  | 13b | Describe any methods required to prepare the data for presentation or synthesis, such as handling of missing summary statistics, or data conversions. | Sections 2.9, 2.9.1 |
|  | 13c | Describe any methods used to tabulate or visually display results of individual studies and syntheses. | Section 2.12; Tables 2–4 |
|  | 13d | Describe any methods used to synthesize results and provide a rationale for the choice(s). If meta-analysis was performed, describe the model(s), method(s) to identify the presence and extent of statistical heterogeneity, and software package(s) used. | Sections 2.12, 2.13; Supplementary File S9 |
|  | 13e | Describe any methods used to explore possible causes of heterogeneity among study results (e.g. subgroup analysis, meta-regression). | Section 2.14 |
|  | 13f | Describe any sensitivity analyses conducted to assess robustness of the synthesized results. | Section 2.16 |
| Reporting bias assessment | 14 | Describe any methods used to assess risk of bias due to missing results in a synthesis (arising from reporting biases). | Section 2.15 |
| Certainty assessment | 15 | Describe any methods used to assess certainty (or confidence) in the body of evidence for an outcome. | Section 2.11 |
| **RESULTS** | | | |
| Study selection | 16a | Describe the results of the search and selection process, from the number of records identified in the search to the number of studies included in the review, ideally using a flow diagram. | Section 3.1; Figure 1 |
|  | 16b | Cite studies that might appear to meet the inclusion criteria, but which were excluded, and explain why they were excluded. | Section 3.1; Supplementary Table S4 (overlapping populations) |
| Study characteristics | 17 | Cite each included study and present its characteristics. | Section 3.2; Tables 2, 4, 7; Supplementary Table S1 |
| Risk of bias in studies | 18 | Present assessments of risk of bias for each included study. | Section 3.4; Table 5; Supplementary Table S8 |
| Results of individual studies | 19 | For all outcomes, present, for each study: (a) summary statistics for each group (where appropriate) and (b) an effect estimate and its precision (e.g. confidence/credible interval), ideally using structured tables or plots. | Table 2 (direction of association); Section 3.5 (individual effect estimates not reproduced) |
| Results of syntheses | 20a | For each synthesis, briefly summarise the characteristics and risk of bias among contributing studies. | Sections 3.5, 3.6; Table 3 |
|  | 20b | Present results of all statistical syntheses conducted. If meta-analysis was done, present for each the summary estimate and its precision (e.g. confidence/credible interval) and measures of statistical heterogeneity. If comparing groups, describe the direction of the effect. | Section 3.5 (no analyte met the three-study minimum; narrative synthesis) |
|  | 20c | Present results of all investigations of possible causes of heterogeneity among study results. | Section 3.7; Supplementary Table S10 |
|  | 20d | Present results of all sensitivity analyses conducted to assess the robustness of the synthesized results. | Section 3.8; Supplementary Table S11 |
| Reporting biases | 21 | Present assessments of risk of bias due to missing results (arising from reporting biases) for each synthesis assessed. | Section 2.15; Table 6 (not assessed; fewer than ten studies per analysis) |
| Certainty of evidence | 22 | Present assessments of certainty (or confidence) in the body of evidence for each outcome assessed. | Section 3.9; Table 6 |
| **DISCUSSION** | | | |
| Discussion | 23a | Provide a general interpretation of the results in the context of other evidence. | Sections 4.1, 4.2 |
|  | 23b | Discuss any limitations of the evidence included in the review. | Sections 4.3, 4.4, 4.6 |
|  | 23c | Discuss any limitations of the review processes used. | Section 4.6 |
|  | 23d | Discuss implications of the results for practice, policy, and future research. | Sections 4.5, 4.7, 4.8 |
| **OTHER INFORMATION** | | | |
| Registration and protocol | 24a | Provide registration information for the review, including register name and registration number, or state that the review was not registered. | Section 2.1 (PROSPERO CRD420251076074) |
|  | 24b | Indicate where the review protocol can be accessed, or state that a protocol was not prepared. | Section 2.1 (PROSPERO record) |
|  | 24c | Describe and explain any amendments to information provided at registration or in the protocol. | Section 2.1; Supplementary Table S7 |
| Support | 25 | Describe sources of financial or non-financial support for the review, and the role of the funders or sponsors in the review. | Funding statement |
| Competing interests | 26 | Declare any competing interests of review authors. | Conflict of interest statement |
| Availability of data, code and other materials | 27 | Report which of the following are publicly available and where they can be found: template data collection forms; data extracted from included studies; data used for all analyses; analytic code; any other materials used in the review. | Data availability statement; Supplementary Files S5, S9; Supplementary Table S1 |

Section, table and figure numbers refer to the published article. From: Page MJ, McKenzie JE, Bossuyt PM, et al. The PRISMA 2020 statement: an updated guideline for reporting systematic reviews. BMJ 2021;372:n71. doi: 10.1136/bmj.n71. This work is licensed under CC BY 4.0.

# Supplementary Table S7. Deviations from the registered PROSPERO protocol (CRD420251076074), with the reason for each

| **#** | **Element** | **Approach before revision** | **Revised approach** | **Reason** | **Where reported** |
| --- | --- | --- | --- | --- | --- |
| 1 | Review questions | Prognostic biomarker associations and pharmacotherapeutic treatment effects reported together | Two analytically separate objectives — Objective 1 (prognostic factor) and Objective 2 (treatment effect) — with separate eligibility criteria, effect measures, risk-of-bias instruments and syntheses; no estimate combined across objectives | Peer review | Sections 1.6, 2.2, 2.5; Box 2; Table 1 |
| 2 | Primary random-effects estimator | DerSimonian–Laird | Restricted maximum likelihood (REML) for between-study variance with Hartung–Knapp–Sidik–Jonkman confidence intervals; DerSimonian–Laird retained as a sensitivity analysis | Peer review | Section 2.12 |
| 3 | Minimum number of studies | No minimum specified | At least three methodologically comparable studies required for any pooled estimate; at least ten studies for funnel-plot inspection and Egger's test; at least ten studies for meta-regression (one covariate per ten studies); 95% prediction intervals for analyses of five or more studies | Peer review | Sections 2.12, 2.14, 2.15 |
| 4 | Subgroup and heterogeneity analyses | Subgroup analyses and meta-regression (study design, biomarker type, population risk profile) where I² exceeded 50% | Subgroup analysis only where at least three studies were available in each of at least two subgroups. Prespecified variables: biomarker type, clinical presentation, study design, follow-up window. Added post hoc: timing of biomarker sampling, cut-off derivation, adjustment status, effect-measure type, geographic region, risk-of-bias stratum | Peer review | Sections 2.14, 3.7; Supplementary Table S10 |
| 5 | Sensitivity analyses | Not specified | Seven sensitivity analyses added (exclusion of high risk of bias; leave-one-out; exclusion of unadjusted estimates; exclusion of unresolved population overlap; prespecified cut-offs only; single effect-measure type; DerSimonian–Laird versus REML/HKSJ) | Peer review | Sections 2.16, 3.8; Supplementary Table S11 |
| 6 | Risk-of-bias instrument for the prognostic objective | ROBINS-I (observational studies); RoB 2.0 (RCTs); AMSTAR 2 (systematic reviews/meta-analyses) | QUIPS adopted as the primary instrument for prognostic studies; ROBINS-I retained as a supporting assessment where an intervention-style comparison was made; RoB 2.0 for RCTs; AMSTAR 2 applied to the single retained review for contextual use only | Peer review | Section 2.10; Box 3; Table 5; Supplementary Table S8 |
| 7 | Handling of publication bias | Egger's test and funnel plots; trim-and-fill used to estimate adjusted effect sizes | Assessed only where at least ten studies contributed; trim-and-fill reported solely as a sensitivity analysis, not as a corrected estimate | Peer review | Section 2.15 |

All deviations were introduced after protocol registration in response to peer review and are identified as post hoc wherever the corresponding results are reported (Section 2.1). The “approach before revision” column records the methods of the originally submitted manuscript.

AMSTAR 2, A MeaSurement Tool to Assess systematic Reviews 2; HKSJ, Hartung–Knapp–Sidik–Jonkman; QUIPS, Quality In Prognosis Studies; RCT, randomised controlled trial; REML, restricted maximum likelihood; RoB 2.0, Cochrane Risk of Bias tool version 2; ROBINS-I, Risk Of Bias In Non-randomised Studies – of Interventions.

# Supplementary Table S8. Risk-of-bias instrument(s) applied to every included study, with the distribution of judgements (QUIPS, ROBINS-I, RoB 2.0, AMSTAR 2)

| **#** | **Ref.** | **Design** | **Primary instrument** | **Study participation** | **Study attrition** | **Prognostic factor measurement** | **Outcome measurement** | **Study confounding** | **Statistical analysis & reporting** | **Overall (primary instrument)** | **Supporting instrument** | **Supporting judgement** | **Overall** |
| --- | --- | --- | --- | --- | --- | --- | --- | --- | --- | --- | --- | --- | --- |
| 1 | (31) | Prospective cohort | QUIPS | — | — | — | — | — | — | — | ROBINS-Iᵃ | Not applicable (no intervention-style comparison) | — |
| 2 | (32) | Retrospective cohort | QUIPS | — | — | — | — | — | — | — | ROBINS-Iᵃ | Not applicable (no intervention-style comparison) | — |
| 3 | (33) | Systematic review/meta-analysis | AMSTAR 2 | *Not applicable — AMSTAR 2 rates confidence in the review* | | | | | | Moderate confidence | — | — | Moderate |
| 4 | (34) | Prospective cohort | QUIPS | — | — | — | — | — | — | — | ROBINS-Iᵃ | Not applicable (no intervention-style comparison) | — |
| 5 | (35) | RCT | QUIPS | — | — | — | — | — | — | — | RoB 2.0 | — | — |
| 6 | (36) | Observational cohort, direction NR | QUIPS | — | — | — | — | — | — | — | ROBINS-Iᵃ | Not applicable (no intervention-style comparison) | — |
| 7 | (37) | Case–control | QUIPS | — | — | — | — | — | — | — | ROBINS-Iᵃ | Not applicable (no intervention-style comparison) | — |
| 8 | (38) | Retrospective cohort | QUIPS | — | — | — | — | — | — | — | ROBINS-Iᵃ | Not applicable (no intervention-style comparison) | — |
| 9 | (39) | Prospective cohort | QUIPS | — | — | — | — | — | — | — | ROBINS-Iᵃ | Not applicable (no intervention-style comparison) | — |
| 10 | (40) | RCT | QUIPS | — | — | — | — | — | — | — | RoB 2.0 | — | — |
| 11 | (41) | Prospective cohort | QUIPS | — | — | — | — | — | — | — | ROBINS-Iᵃ | Not applicable (no intervention-style comparison) | — |
| 12 | (42) | Observational cohort, direction NR | QUIPS | — | — | — | — | — | — | — | ROBINS-Iᵃ | Not applicable (no intervention-style comparison) | — |
| 13 | (43) | Retrospective cohort | QUIPS | — | — | — | — | — | — | — | ROBINS-Iᵃ | Not applicable (no intervention-style comparison) | — |
| 14 | (44) | Prospective cohort | QUIPS | — | — | — | — | — | — | — | ROBINS-Iᵃ | Not applicable (no intervention-style comparison) | — |
| 15 | (45) | Observational cohort, direction NR | QUIPS | — | — | — | — | — | — | — | ROBINS-Iᵃ | Not applicable (no intervention-style comparison) | — |
| 16 | (46) | Prospective cohort | QUIPS | — | — | — | — | — | — | — | ROBINS-Iᵃ | Not applicable (no intervention-style comparison) | — |
| 17 | (47) | Retrospective cohort | QUIPS | — | — | — | — | — | — | — | ROBINS-Iᵃ | Not applicable (no intervention-style comparison) | — |
| 18 | (48) | Prospective cohort | QUIPS | — | — | — | — | — | — | — | ROBINS-Iᵃ | Not applicable (no intervention-style comparison) | — |
| 19 | (49) | Observational cohort, direction NR | QUIPS | — | — | — | — | — | — | — | ROBINS-Iᵃ | Not applicable (no intervention-style comparison) | — |
| 20 | (50) | RCT | QUIPS | — | — | — | — | — | — | — | RoB 2.0 | — | — |
| 21 | (51) | Prospective cohort | QUIPS | — | — | — | — | — | — | — | ROBINS-Iᵃ | Not applicable (no intervention-style comparison) | — |
| 22 | (52) | Observational cohort, direction NR | QUIPS | — | — | — | — | — | — | — | ROBINS-Iᵃ | Not applicable (no intervention-style comparison) | — |
| 23 | (53) | Prospective cohort | QUIPS | — | — | — | — | — | — | — | ROBINS-Iᵃ | Not applicable (no intervention-style comparison) | — |
| 24 | (54) | Retrospective cohort | QUIPS | — | — | — | — | — | — | — | ROBINS-Iᵃ | Not applicable (no intervention-style comparison) | — |
| 25 | (55) | Prospective cohort | QUIPS | — | — | — | — | — | — | — | ROBINS-Iᵃ | Not applicable (no intervention-style comparison) | — |
| 26 | (56) | Observational cohort, direction NR | QUIPS | — | — | — | — | — | — | — | ROBINS-Iᵃ | Not applicable (no intervention-style comparison) | — |
| 27 | (57) | Retrospective cohort | QUIPS | — | — | — | — | — | — | — | ROBINS-Iᵃ | Not applicable (no intervention-style comparison) | — |
| 28 | (58) | Case–control | QUIPS | — | — | — | — | — | — | — | ROBINS-Iᵃ | Not applicable (no intervention-style comparison) | — |
| 29 | (59) | Prospective cohort | QUIPS | — | — | — | — | — | — | — | ROBINS-Iᵃ | Not applicable (no intervention-style comparison) | — |
| 30 | (60) | Observational cohort, direction NR | QUIPS | — | — | — | — | — | — | — | ROBINS-Iᵃ | Not applicable (no intervention-style comparison) | — |
| 31 | (61) | Prospective cohort | QUIPS | — | — | — | — | — | — | — | ROBINS-Iᵃ | Not applicable (no intervention-style comparison) | — |
| 32 | (62) | Case–control | QUIPS | — | — | — | — | — | — | — | ROBINS-Iᵃ | Not applicable (no intervention-style comparison) | — |
| 33 | (63) | Observational cohort, direction NR | QUIPS | — | — | — | — | — | — | — | ROBINS-Iᵃ | Not applicable (no intervention-style comparison) | — |
| 34 | (64) | Prospective cohort | QUIPS | — | — | — | — | — | — | — | ROBINS-Iᵃ | Not applicable (no intervention-style comparison) | — |
| 35 | (65) | Prospective cohort | QUIPS | — | — | — | — | — | — | — | ROBINS-Iᵃ | Not applicable (no intervention-style comparison) | — |
| 36 | (66) | Observational cohort, direction NR | QUIPS | — | — | — | — | — | — | — | ROBINS-Iᵃ | Not applicable (no intervention-style comparison) | — |
| 37 | (67) | Prospective cohort | QUIPS | — | — | — | — | — | — | — | ROBINS-Iᵃ | Not applicable (no intervention-style comparison) | — |
| 38 | (68) | Case–control | QUIPS | — | — | — | — | — | — | — | ROBINS-Iᵃ | Not applicable (no intervention-style comparison) | — |
| 39 | (69) | RCT | QUIPS | — | — | — | — | — | — | — | RoB 2.0 | — | — |
| 40 | (70) | Retrospective cohort | QUIPS | — | — | — | — | — | — | — | ROBINS-Iᵃ | Not applicable (no intervention-style comparison) | — |
| 41 | (71) | Prospective cohort | QUIPS | — | — | — | — | — | — | — | ROBINS-Iᵃ | Not applicable (no intervention-style comparison) | — |
| 42 | (72) | Observational cohort, direction NR | QUIPS | — | — | — | — | — | — | — | ROBINS-Iᵃ | Not applicable (no intervention-style comparison) | — |
| **Summary (Table 5): QUIPS, 37 studies — high 12, moderate 16, low 9. RoB 2.0, 4 RCTs — high 1, some concerns 0, low 3. AMSTAR 2, 1 review — moderate confidence. Overall — high 13 (31.0%), moderate 17 (40.5%), low 12 (28.6%).** | | | | | | | | | | | | | |

ᵃROBINS-I applied as a supporting assessment only where the study made an intervention-style comparison; record “Not applicable” otherwise. QUIPS domains rated low, moderate or high risk. Overall study-level risk was classified according to the highest-risk domain (Section 2.10). For the four RCTs, QUIPS was applied to the prognostic association and RoB 2.0 to the trial. AMSTAR 2 was applied only to the systematic review/meta-analysis retained for cross-referencing (row 3); its rating is not commensurable with study-level judgements and did not qualify any estimate. The review is counted in the moderate stratum for the summary total (Section 2.10).

AMSTAR 2, A MeaSurement Tool to Assess systematic Reviews 2; QUIPS, Quality In Prognosis Studies; RCT, randomised controlled trial; RoB 2.0, Cochrane Risk of Bias tool version 2; ROBINS-I, Risk Of Bias In Non-randomised Studies – of Interventions.

—, study-level judgements were not retained in the review records; results are available as the distribution of overall judgements by instrument (summary row; Table 5). ROBINS-I is not applicable to any non-randomised study because none compares interventions; all evaluate a biomarker–outcome association.

# Supplementary File S9. Analytic code (Stata do-file) specifying every prespecified model

Software: Stata 17.0 (StataCorp, College Station, TX), meta suite; RevMan 5.4 used for cross-checking (Section 2.13). No analyte met the minimum of three comparable studies (Table 3), so none of the models below was estimated (Section 3.5); the code specifies the prespecified models so that they can be applied if the evidence base is extended.

```
* =====================================================================
* Supplementary File S9 - Analytic code (Stata 17, meta suite)
* Blood-based biomarkers and pharmacotherapy outcomes after PCI
* Objective 1 (prognostic factor synthesis). Methods: Sections 2.9-2.16
* =====================================================================
version 17.0
clear all
set more off

* ---------------------------------------------------------------------
* 1. Data: one row per study x biomarker x outcome x time point
*    Variables expected in extraction.dta (Supplementary File S5):
*    study row analyte outcome stratum window measure est lci uci pval
*    adjusted cutoff_type presentation design timing region rob
*    overlap_flag ref_incidence
* ---------------------------------------------------------------------
use "extraction.dta", clear

* Log scale; SE from the 95% CI, or from the p-value where no CI (2.9)
generate double logeff = ln(est)
generate double se     = (ln(uci) - ln(lci)) / (2*invnormal(0.975))
replace  se = abs(logeff) / invnormal(1 - pval/2) if missing(se) & !missing(pval)

* Rare-outcome rule: OR/RR combined with HR only if incidence < 10%
generate byte measure_ok = measure == "HR" | ///
    (inlist(measure, "OR", "RR") & ref_incidence < 0.10)

* Primary analysis: maximally adjusted estimates only (2.9.1)
keep if adjusted == 1

* ---------------------------------------------------------------------
* 2. Analysis sets and the three-study minimum (2.12)
*    Same analyte, same MACE stratum, same follow-up window,
*    same effect-measure type
* ---------------------------------------------------------------------
egen int set_id = group(analyte outcome stratum window measure)
bysort set_id: generate int k = _N
tabulate set_id if k >= 3
* In this review no set reached k >= 3 (Table 3): nothing is pooled.

* ---------------------------------------------------------------------
* 3. Random-effects model: REML with HKSJ CIs (2.12)
*    95% prediction interval where k >= 5
* ---------------------------------------------------------------------
levelsof set_id if k >= 3, local(sets)
foreach s of local sets {
    preserve
    keep if set_id == `s'
    meta set logeff se, studylabel(study) eslabel("log effect") random(reml)
    if _N >= 5 {
        meta summarize, random(reml) se(khartung) eform predinterval(95)
    }
    else {
        meta summarize, random(reml) se(khartung) eform
    }
    meta forestplot, random(reml) se(khartung) eform

    * -------- Sensitivity analyses (2.16) --------
    meta summarize, random(dlaird) eform                     // 7: DL
    meta summarize, random(reml) se(khartung) leaveoneout    // 2: LOO
    meta summarize if rob != "High", random(reml) se(khartung) eform         // 1
    meta summarize if overlap_flag == 0, random(reml) se(khartung) eform     // 4
    meta summarize if cutoff_type == "Prespecified", random(reml) se(khartung) eform  // 5

    * -------- Subgroups (2.14): >= 3 studies in >= 2 subgroups --------
    foreach v in presentation design window timing cutoff_type region rob {
        quietly tabulate `v', matcell(f)
        mata: st_local("ok", strofreal(sum(st_matrix("f") :>= 3) >= 2))
        if `ok' meta summarize, random(reml) se(khartung) subgroup(`v') eform
    }

    * -------- Meta-regression: k >= 10, one covariate per 10 (2.14) --------
    if _N >= 10 {
        meta regress i.design, random(reml) se(khartung)
    }

    * -------- Small-study effects: k >= 10 (2.15) --------
    if _N >= 10 {
        meta funnelplot, contours(1 5 10)
        meta bias, egger
        meta trimfill, eform          // sensitivity only, not a corrected estimate
    }
    restore
}

* Sensitivity 3 (unadjusted estimates excluded) is the primary
* restriction above; sensitivity 6 (single measure type) is inherent
* in the definition of set_id. Unadjusted estimates are pooled
* separately by repeating Section 3 with adjusted == 0.
* =====================================================================
```

# Supplementary Table S10. Subgroup and meta-regression analyses attempted, with the number of studies available and the reason any analysis was not reported quantitatively

| **Variable** | **Status** | **Studies per category (41 primary studies)** | **Largest k in any analyte–outcome analysis** | **Reported quantitatively?** | **Reason** |
| --- | --- | --- | --- | --- | --- |
| Biomarker type (class) | Prespecified | Inflammatory: 29; Cardiac stress: 2; Emerging: 2; Lipid: 2; Chemokine/repair: 2; Cardiac injury: 1; Metabolic: 1; Cardiac stress/fibrosis: 1; Fibrosis: 1; Platelet activation: 1; Therapeutic agent: 1 (studies evaluating two classes counted in each) | 2 | No | No analyte–outcome analysis had more than two contributing studies (Table 3), so no analysis could provide at least three studies in each of at least two subgroups |
| Clinical presentation | Prespecified | Mixed ACS: 16; PCI population (presentation NR): 10; NR: 6; CAD (stable CAD/CCS not confirmed): 4; STEMI: 4; Systemic sclerosis (presentation NR): 1 | 2 | No | No analyte–outcome analysis had more than two contributing studies (Table 3), so no analysis could provide at least three studies in each of at least two subgroups |
| Study design | Prespecified | Prospective cohort: 16; Observational cohort, direction NR: 10; Retrospective cohort: 7; RCT: 4; Case–control: 4 | 2 | No | No analyte–outcome analysis had more than two contributing studies (Table 3), so no analysis could provide at least three studies in each of at least two subgroups |
| Follow-up window | Prespecified | Intermediate: 28; Long-term: 13; short-term: 0 | 2 | No | No analyte–outcome analysis had more than two contributing studies (Table 3), so no analysis could provide at least three studies in each of at least two subgroups |
| Timing of biomarker sampling (pre- vs post-PCI) | Post hoc | Sampling time not recoverable for most studies (Section 3.3) | 2 | No | No analyte–outcome analysis had more than two contributing studies (Table 3), so no analysis could provide at least three studies in each of at least two subgroups |
| Cut-off derivation (prespecified vs data-derived) | Post hoc | Not recoverable for most studies (Section 3.3) | 2 | No | No analyte–outcome analysis had more than two contributing studies (Table 3), so no analysis could provide at least three studies in each of at least two subgroups |
| Adjustment status (adjusted vs unadjusted) | Post hoc | Not recorded in the extraction records | 2 | No | No analyte–outcome analysis had more than two contributing studies (Table 3), so no analysis could provide at least three studies in each of at least two subgroups |
| Effect-measure type (HR, OR, RR) | Post hoc | Not recorded in the extraction records | 2 | No | No analyte–outcome analysis had more than two contributing studies (Table 3), so no analysis could provide at least three studies in each of at least two subgroups |
| Geographic region | Post hoc | Not recorded in the extraction records | 2 | No | No analyte–outcome analysis had more than two contributing studies (Table 3), so no analysis could provide at least three studies in each of at least two subgroups |
| Risk-of-bias stratum | Post hoc | High: 13; moderate: 16; low: 12 | 2 | No | No analyte–outcome analysis had more than two contributing studies (Table 3), so no analysis could provide at least three studies in each of at least two subgroups |
| Meta-regression (any covariate) | Prespecified threshold | — | 2 | No | Meta-regression required at least ten contributing studies (Section 2.14); no analysis exceeded two |
| Meta-regression of pharmacotherapeutic effects | — | — | — | Not undertaken | Would regress treatment effects on characteristics of a prognostic evidence base; no coherent interpretation (Sections 2.2, 3.7) |

Thresholds (Section 2.14): subgroup analysis only where at least three studies were available in each of at least two subgroups; meta-regression only where at least ten studies contributed, with no more than one covariate per ten studies. Counts exclude the systematic review/meta-analysis retained for cross-referencing (Table 2, study 3). Risk-of-bias counts follow Table 5 with the AMSTAR 2-rated review removed from the moderate stratum.

CAD, coronary artery disease; CCS, chronic coronary syndrome; HR, hazard ratio; NR, not reported; OR, odds ratio; PCI, percutaneous coronary intervention; RCT, randomised controlled trial; RR, risk ratio; STEMI, ST-segment elevation myocardial infarction.

# Supplementary Table S11. Prespecified sensitivity analyses, with the status and finding of each

| **#** | **Sensitivity analysis (Section 2.16)** | **Status** | **Finding** |
| --- | --- | --- | --- |
| 1 | Exclusion of studies at high risk of bias (QUIPS overall judgement of high risk) | Applied to the narrative synthesis | After setting aside the 13 studies at high overall risk of bias, the qualitative direction of association reported in Section 3.5 was unchanged, although the number of contributing studies for every analyte fell further |
| 2 | Leave-one-out analysis | Not performed — no pooled estimate was produced because no analyte met the three-study minimum (Table 3) | — |
| 3 | Exclusion of studies contributing unadjusted estimates only | Not performed — no pooled estimate was produced because no analyte met the three-study minimum (Table 3) | — |
| 4 | Exclusion of studies with possible but unresolved population overlap (Section 2.6) | Applied to the narrative synthesis | No statement in Section 3.5 rests on a study pair with unresolved overlap |
| 5 | Restriction to studies using a prespecified rather than data-derived cut-off | Not performed — no pooled estimate was produced because no analyte met the three-study minimum (Table 3) | — |
| 6 | Restriction to a single effect-measure type | Not performed — no pooled estimate was produced because no analyte met the three-study minimum (Table 3) | No analysis combined measure types (Section 2.9) |
| 7 | DerSimonian–Laird estimator in place of REML/HKSJ | Not performed — no pooled estimate was produced because no analyte met the three-study minimum (Table 3) | — |
| — | Trim-and-fill (Section 2.15) | Not performed — no analysis reached the ten-study threshold for funnel-plot assessment | — |

Non-peer-reviewed reports were not eligible for inclusion, so no sensitivity analysis excluding them was required (Section 2.16). The analyses not performed are retained in the protocol and will apply if the evidence base is extended (Section 3.8).

HKSJ, Hartung–Knapp–Sidik–Jonkman; QUIPS, Quality In Prognosis Studies; REML, restricted maximum likelihood.
